# UFL1 promotes histone H4 ufmylation and ATM activation

**DOI:** 10.1038/s41467-019-09175-0

**Published:** 2019-03-18

**Authors:** Bo Qin, Jia Yu, Somaira Nowsheen, Minghui Wang, Xinyi Tu, Tongzheng Liu, Honglin Li, Liewei Wang, Zhenkun Lou

**Affiliations:** 1 Department of Oncology, Mayo Clinic, Rochester, MN 55905 USA; 2 Division of Clinical Pharmacology, Department of Molecular Pharmacology and Experimental Therapeutics, Mayo Clinic, Rochester, MN 55905 USA; 3 Mayo Medical Scientist Training Program, Mayo Medical School and Mayo Graduate School, Mayo Clinic, Rochester, MN 55905 USA; 4 Department of Genetics and Genomic Sciences, Icahn Institute of Genomics and Multiscale Biology, Icahn School of Medicine at Mount Sinai, 1470 Madison Avenue, New York, NY 10029 USA; 5 Institute of Tumor Pharmacology, Jinan University, 510632 Guangzhou, China; 6 Department of Biochemistry & Molecular Biology, Cancer Center, Georgia Regents University, Augusta, GA 30912 USA

## Abstract

The ataxia-telangiectasia mutated (ATM) kinase, an upstream kinase of the DNA damage response (DDR), is rapidly activated following DNA damage, and phosphorylates its downstream targets to launch DDR signaling. However, the mechanism of ATM activation is still not completely understood. Here we report that UFM1 specific ligase 1 (UFL1), an ufmylation E3 ligase, is important for ATM activation. UFL1 is recruited to double strand breaks by the MRE11/RAD50/NBS1 complex, and monoufmylates histone H4 following DNA damage. Monoufmylated histone H4 is important for Suv39h1 and Tip60 recruitment. Furthermore, ATM phosphorylates UFL1 at serine 462, enhancing UFL1 E3 ligase activity and promoting ATM activation in a positive feedback loop. These findings reveal that ufmylation of histone H4 by UFL1 is an important step for amplification of ATM activation and maintenance of genomic integrity.

## Abstract (web summary)

Ufmylation is a ubiquitylation-like protein modification but only a few ufmylation substrates and functions have been discovered so far. Here, the authors demonstrate ufmylation of histone H4 upon DNA damage and show that this modification is involved in the amplification of ATM activation.

## Introduction

When DNA double-strand break (DSB) occurs, rapid DNA damage response (DDR) and DNA repair are required to preserve genome integrity^1^. The protein kinase ataxia-telangiectasia mutated (ATM) functions as an apical activator for the whole process, and controls signaling and the DNA repair network^2,3^. Germline mutations of the *ATM* gene tend to destabilize ATM protein and cause ataxia-telangiectasia (AT) syndrome/Louis–Bar syndrome. AT is a rare, neurodegenerative, and autosomal recessive disease-causing severe disability. AT patients display immunodeficiency, radiosensitivity, progressive cerebellar ataxia, and cancer susceptibility and commonly develop neurodegenerative disease, metabolic syndrome and cancer^4,5^.

The MRE11–RAD50–NBS1 (MRN) complex is important for activation of ATM kinase^6–9^. Activated ATM phosphorylates histone H2AX at Ser139 (γH2AX) close to DNA damage sites, and then recruits MDC1, which serves as a platform for binding more MRN complexes and other DNA repair proteins to amplify DDR signaling and promote DNA repair^10–15^. In addition, ATM activation is also dependent on the acetyltransferase Tip60. Tip60 is recruited to the sites of DNA damage by binding to H3K9me3, and in turn acetylates ATM at lysine 3016 and boosts ATM autophosphorylation and activation^16,17^. Tip60 itself is phosphorylated by c-Abl, which increases Tip60 activity and reinforces ATM activity^18^. However, early chromatin context leading to full ATM activation remains unclear.

Post-translational modification is critical for ATM activation. In addition to phosphorylation, acetylation and methylation, ubiquitination is also important for ATM activation. Skp2 mediated NBS1 ubiquitination enhances the interaction between NBS1 and ATM and promotes ATM activation^19^. CHFR and RNF8 are also found to synergistically regulate histone H2B ubiquitination and chromatin relaxation, and promote ATM activation^20^. In ubiquitination reaction, three classes of enzymes work orchestrally to add ubiquitin to the substrate. The first enzyme, E1, consecutively thioesterificates and activates the C terminus of ubiquitin. E1 then transfers ubiquitin to the cysteine side chain in the second enzyme E2. At last, the E2 and E3 enzymes together transfer the ubiquitin (Ub) from the E2 enzyme to the substrate^21^. Conversely, ubiquitin conjugation could be removed by ubiquitin protease in a reaction called deubiquitination. In addition to ubiquitin, other Ub like polypeptides such as SUMO and NEDD8 could be conjugated to target proteins through E1-E2-E3 catalyzed reactions.

Recently, ubiquitin-fold modifier1 (UFM1) – a new ubiquitin-like protein was identified^22^. UFM1 conjugation system is a ubiquitin-like modification system including UBA5 (E1-like), UFC1 (E2-like), and UFL1 (E3-like)^22,23^. Thus far, UFL1 is the only known E3 ligase that has been discovered to conjugate UFM1 to its substrates^23^. Similar to deubiquitination, UFM1 can be removed from substrates by UFM1-specific proteases (UFSP). Till now, only one functional UFSP protein called UFSP2 has been identified in human^24^. The ufmylation system is less explored. So far, it has only been discovered in animals and plants and only two substrates have been determined- UFBP1 and ASC1^22,25^. Previous studies have demonstrated the important roles of ufmylation in erythroid development, breast cancer development, and protecting pancreatic beta cells from ER stress-induced apoptosis^25–28^.

Here we reveal a role of ufmylation in the DDR and identify histone H4 as a substrate of the ufmylation system. We find that MRN complex recruits UFL1 to DSBs and UFL1 ufmylated histone H4 at lysine 31, enhancing recruitment of Suv39h1 complex to the DSBs. Suv39h1 then trimethylates histone H3 at lysine 9 around DSBs, recruiting Tip60 and promoting ATM activation. Our findings suggest that ufmylation is important for amplification of ATM activation, thus maintaining genomic integrity.

## Results

### Recruitment of UFL1 to DSBs by the MRN complex

**Fig. 1:**
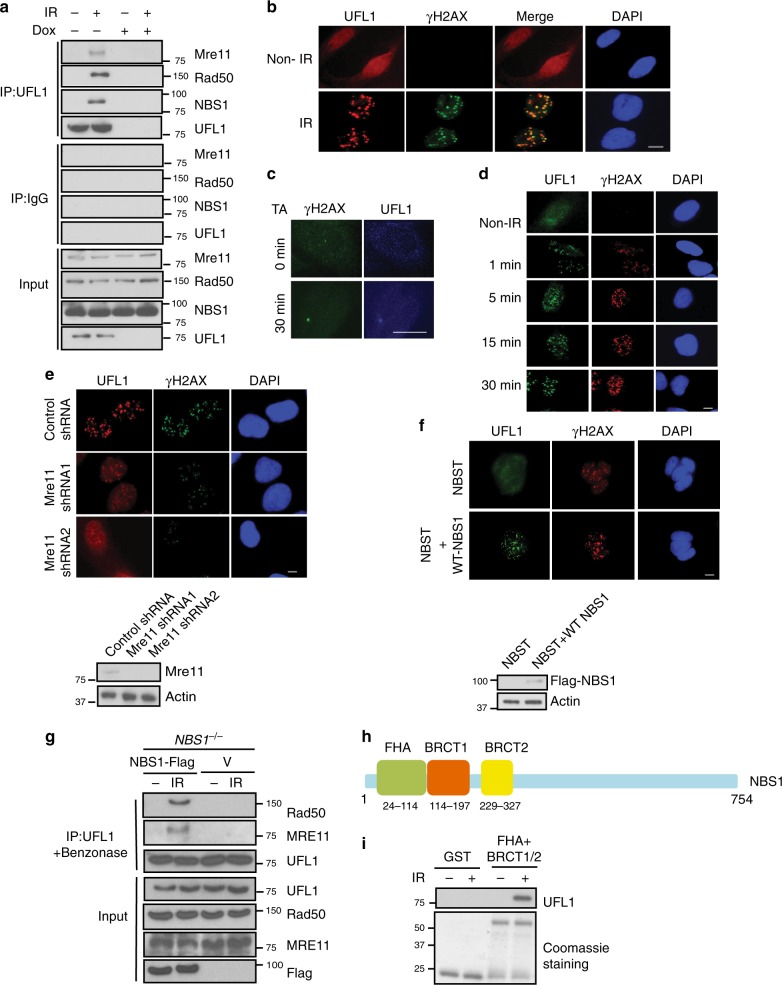
UFL1 protein accumulates at DSBs through the MRN complex. **a** U2OS cells stably expressing UFL1 Tet-on shRNA were treated with doxycycline (Dox) for 3–5 days, and then treated with or without 2 Gy IR. After 30 min, cells were harvested and lysed with NETN buffer. Cell lysates were incubated with UFL1 antibody + Benzonase. The immunoprecipitates were blotted with indicated antibodies. **b** Immunofluorescence of UFL1 and γH2AX in U2OS cells irradiated with IR (0.5 Gy). **c** Triamcinolone acetonide (TA) treatment induces the translocation of RFP-I-SceI-GR fusion protein from the cytoplasm to the nucleus and generates one double strand break at the cutting site. The protein localization was detected by indicated antibodies. **d** UFL1 foci formation at different time points following 0.5 Gy IR treatment. **e**, **f** UFL1 foci formation was analyzed in Mre11 knockdown cells or *NBS1* deficient cells (NBST). **g** NBST cells were transfected with Vector (V) or Flag-NBS1 and treated with or without 2 Gy IR. After 30 min, the cells were lysed and immunoprecipitation with UFL1 antibody with Benzonase treatment was performed. The immunoprecipitates were blotted with indicated antibodies. **h** The schematic diagram of NBS1 protein domain. **i** U2OS cells were treated with or without 2 Gy IR, and the cell lysates were pulled down with GST, GST-NBS1 FHA+BRCT (1+2) proteins. After washes, the beads were boiled and analyzed with indicated antibodies. Scale bars, 10 µm. Source data are provided as a Source Data file

When we studied cellular function of UFL1, we unexpectedly found that UFL1 interacted with the MRN complex in a DNA damage-inducible manner (Fig. 1a and Supplementary Figure 1a). These interactions are resistant to Benzonase treatment, suggesting protein–protein interactions are independent of DNA. Due to the important function of the MRN complex in the DDR^29^, we hypothesized that UFL1 might be involved in the DDR. Following DNA damage, various proteins involved in DDR pathways, such as MDC1, BRCA1 and 53BP1^11–13,30–33^, aggregate at DNA lesions and help DDR signaling and DNA repair. Hence, we first tested whether UFL1 localized to the sites of DNA damage. Under unstressed condition, UFL1 localized in the cytoplasm and nucleus. However, following ionizing irradiation (IR), nuclear UFL1 protein formed discrete nuclear foci and colocalized with γH2AX (Fig. 1b and Supplementary Figure 1b), indicating that UFL1 relocalizes to DNA lesions. To further confirm this finding, we utilized an inducible DSB system, in which addition of triamcinolone acetonide (TA) induced nuclear translocation of I-SceI and created a single DSB in the genomic DNA^34^. We found that UFL1 accumulated at DSB site after TA treatment for 30 min **(**Fig. 1c**)**. UFL1 foci formation was detected as early as 1 min following IR, suggesting that UFL1 is involved in early DDR signaling (Fig. 1d). We next tested how UFL1 was recruited to DNA lesions. Because of its interaction with the MRN complex, we first tested whether the MRN complex was important for UFL1 recruitment. We found that depletion of MRE11/Rad50/NBS1 attenuated UFL1 foci formation (Fig. 1e and Supplementary Figure 1c-e). To further confirm this, we utilized the NBS1 deficient cell line NBST^35^. In NBST cells, UFL1 failed to form nuclear foci following DNA damage, while reconstitution of WT NBS1 resulted in the accumulation of UFL1 to DSBs (Fig. 1f and Supplementary Figure 1f). NBS1 deficiency abolished the interaction between UFL1 and MRN complex (Fig. 1g), suggesting NBS1 might be important for UFL1 recruitment to DSB. We further found that the FHA and BRCT1/2 domains of NBS1 protein were sufficient for its interaction with UFL1 (Fig. 1h, i). However, depletion of UFL1 did not affect the recruitment of NBS1 to DSBs (Supplementary Figure 1g). Taken together, these results suggest that the MRN complex is important for UFL1 recruitment to DSBs.

### Activation of ATM signaling by UFL1

**Fig. 2:**
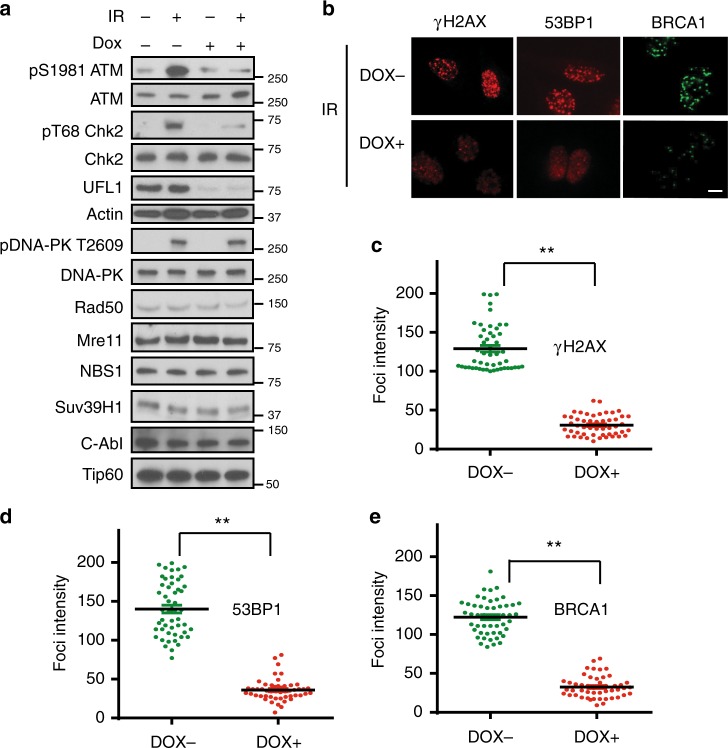
UFL1 regulates the ATM signaling. **a** U2OS cells expressing UFL1 tet-on shRNA1 were irradiated with 2 Gy IR. Thirty minutes later, cells were lysed and blotted with indicated antibodies. **b** Representative picture of γH2AX, 53BP1, and BRCA1 foci in control (Dox-) and UFL1 knockdown (Dox+) U2OS cells 1 h after 0.5 Gy treatment. Scale bars, 10 µm. **c**–**e** Quantification of intensities of γH2AX, 53BP1, and BRCA1 foci in control (Dox-) and UFL1 knockdown (Dox+) U2OS cells. Data presented as mean ± SD of *n* = 50 cells. **p < 0.01 by Student’s *t*-test. Source data are provided as a Source Data file

**Fig. 3:**
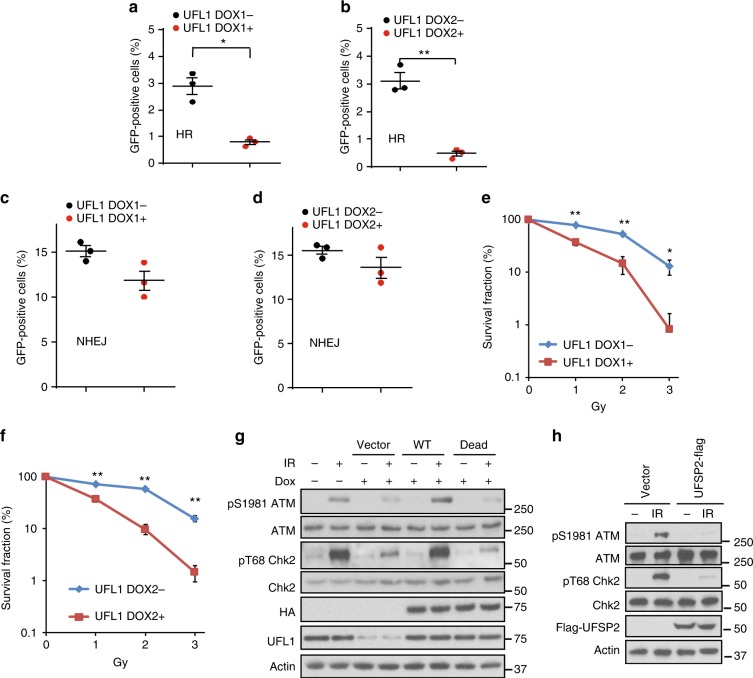
UFL1 regulates the DNA damage response. **a**–**d** U2OS cells integrated with HR or NHEJ reporter and infected with UFL1 Tet-on shRNA1 and shRNA2 virus were treated with doxycycline and subjected to the HR assay and NHEJ assay as described in the method. Data presented as mean±SD of *n* = 3 biological replicates. *p < 0.05, **p < 0.01. Statistical significance was calculated using Student *t*-test. **e**, **f** Colony-formation assay following IR was performed for U2OS cells stably expressing UFL1 Tet-on shRNA1 and shRNA2 with or without doxycycline (Dox). The data presented as mean±s.e.m of *n* = 3 independent experiments. *p < 0.05, **p < 0.01. Statistical significance was calculated using two-way ANOVA. **g** Cells expressing inducible UFL1 shRNA1 and/or reconstituted with shRNA resistant wildtype (WT) and ligase dead mutant Flag-UFL1 (DEAD) were irradiated with 2 Gy IR. Thirty minutes later, cells were lysed and blotted with indicated antibodies. **h** UFSP2-Flag or vector plasmids were transfected into U2OS cells. 24 h later, cells were irradiated with 2 Gy IR, and cell lysates were blotted with indicated antibodies. Source data are provided as a Source Data file

We next examined how UFL1 affects DNA repair and DDR signaling. ATM is the primary transducer of DSB response that is activated in a MRN complex and Tip60 dependent manner^2^. Once ATM is activated, it phosphorylates downstream DDR factors, such as H2AX, MDC1, and Chk2 to initiate DDR signaling^10,36,37^. As UFL1 interacts with the MRN complex, we next tested whether UFL1 regulates ATM activation. By using phosphorylation of ATM at serine 1981 as a functional readout, we found that loss of UFL1 significantly reduced ATM phosphorylation, but not ATM protein level. Loss of UFL1 also reduced IR-induced Chk2 phosphorylation (Fig. 2a and Supplementary Figure 2a). The protein level of other factors involved in ATM activation such as the MRN complex, Tip60, c-Abl, and Suv39h1 were not altered. Histone modifications, including H2BK120ub, H3K79me1, and H3K4me1 were slightly inhibited, but other modifications such as H3K27me2, and H4K20me3 did not change (Supplementary Figure 2b). In addition, DNA-PK phosphorylation in response to IR was not affected (Fig. 2a). As H2AX, BRCA1, and 53BP1 are important factors involved in DSB repair, we next examined γH2AX, 53BP1, and BRCA1 foci formation in UFL1 depleted cells. We found over 90% of cells were γH2AX foci positive in both groups, although the intensity is decreased in UFL1 knockdown cells. We also found decreased BRCA1 and 53BP1 foci intensity in UFL1-depleted cells (Fig. 2b–e, Supplementary Figure. 2c–e). In addition, we found that homologous recombination (HR) but not non-homologous recombination (NHEJ) was compromised in two different UFL1 depleted cells by GFP reporter assay (Fig. 3a–d). The relatively normal NHEJ might be due to normal DNA-PK activation. Cell cycle distribution of UFL1 depleted cells was comparable to control cells (Supplementary Figure 2f, g). Consistent with compromised ATM signaling, cells depleted of UFL1 showed defective G2/M checkpoint and were more sensitive to IR treatment in clonogenic survival assays (Supplementary Figure 2j and Fig. 3e, f), and further knockdown of UFL1 in ATM depleted cells did not further enhance the cell sensitivity to IR treatment (Supplementary Figure 2h, i) or further impair the G2/M checkpoint (Supplementary Figure 2j). Reconstitution of wild-type UFL1 restored ATM and Chk2 phosphorylation (Fig. 3g). We also identified a motif of UFL1 that is similar to a motif of UBA5 that is responsible for UFC1 binding (Supplementary Figure 2k, l). Deletion of this motif (Δ3) abolished UFC1 binding, and when UFL1 depleted cells were reconstituted with the Δ3 UFL1 mutant (UFL1 dead mutant), ATM activation was not restored (Fig. 3g, and Supplementary Figure 2m). We also investigated UFSP2, which functions as a deufmylases to cleave UFM1 chain from its substrates^24^. Overexpression of UFSP2 also suppressed ATM activation (Fig. 3h). These studies suggest that the effect we observed by UFL1 knockdown depends on the interaction between UFL1 and UFC1. We also depleted UFM1 and found that knockdown of UFM1 also inhibited ATM activation (Supplementary Figure 2n–o). Collectively, these results suggest that UFL1 regulates ATM activation.

### Ufmylation of H4 by UFL1 following DNA damage

**Fig. 4:**
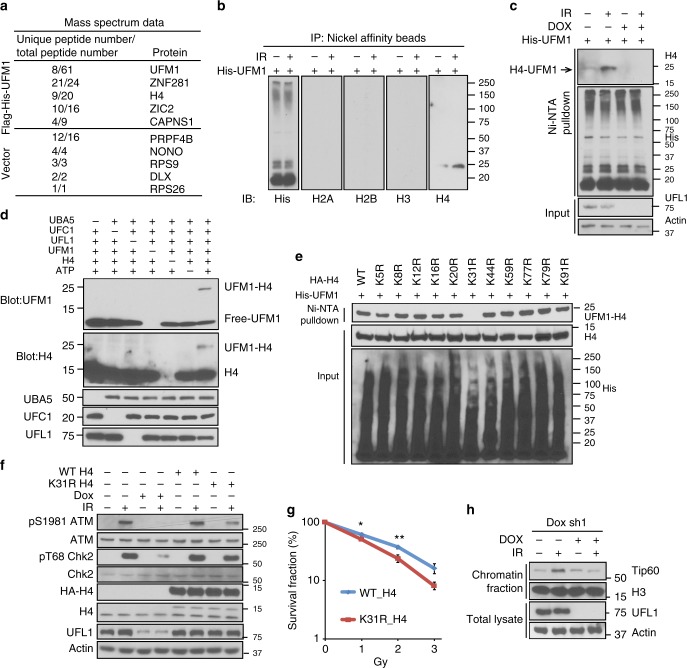
UFL1 monoufmylates histone H4 and promotes ATM activation. **a** Selected proteins identified by mass spectrometry from irradiated 293T cell expressing Flag-His vector or Flag-His-UFM1. *N* = 1 sample in each group was analyzed. The full list of identified proteins is provided in Supplementary Data 1. Among histone proteins, only H4 is enriched in the Flag-His-UFM1 purification (9 unique/20 total peptides) compared to the Flag-His purification (6 unique/7 total peptides), suggesting that H4 might be ufmylated. **b** Flag-His-ufmylated proteins were purified from 293T cells before and after IR (2 Gy) after purification with nickel beads and anti-Flag agarose. The immunoprecipitates were detected with indicated antibodies. **c** Flag-His-ufmylated H4 was purified from control and UFL1 knockdown cells with or without 2 Gy IR and blotted with indicated antibodies. **d** In vitro ufmylation assay. Purified UBA5, UFC1, UFL1, UFM1, and H4 proteins were incubated together in the presence of ATP and MgCl_2_ at 30^ °^C for 90 min. The reaction products were probed with indicated antibodies. **e** Wildtype (WT) histone H4 and 11 different single lysine (K) to arginine (R) mutants were transfected into U2OS cells. Flag and His tandem purification was performed and H4 ufmylation was analyzed. **f** Constructs expressing WT or K31R H4 were transfected into U2OS tet-on UFL1 shRNA expressing cells, and the cells were treated with doxycycline as indicated. Thirty minutes after 2 Gy IR, the cells were harvested and blotted with indicated antibodies. **g** Colony formation of U2OS cells expressing WT H4 or H4K31R following IR. The data presented are mean ± s.e.m. for *n* = 3 independent experiments. Statistical significance was calculated using two-way ANOVA. *p < 0.05, **p < 0.01. **h** U2OS cells stably expressing UFL1 Tet-on shRNA were treated with doxycycline (Dox) and irradiated with 2 Gy IR. Thirty minute later, cells were harvested. Half of the cells were lysed. Chromatin fractions were isolated from other half of cells. The samples were blotted with indicated antibodies. Source data are provided as a Source Data file

We next investigated how ufmylation regulates ATM activation. We initially hypothesized that ATM could be ufmylated following DNA damage. However, we failed to detect ATM ufmylation (Supplementary Figure 3a). To identify the substrates of UFL1 involved in ATM activation, we purified His-UFM1 modified proteins under denaturing conditions. Mass spectrometry data revealed several candidate UFM1 modified substrates, and we eventually focused on histone H4 after some initial testing (Fig. 4a and Supplementary Data 1). To verify the mass spectrometry results, we utilized tandem affinity purification method to purify proteins covalently-linked to His-tagged UFM1, and found histone H4, but not other histones, to be modified by UFM1 and this modification was inducible by DNA damage (Fig. 4b). Furthermore, depletion of UFL1 alleviated H4 ufmylation (Fig. 4c). UFM1 is a ubiquitin like protein and its mature protein molecular weight is ~9 kDa. Judged from the molecular weight of modified histone H4, it is likely to be mono-ufmylated. To determine whether H4 is a genuine substrate of UFL1, we used purified ufmylation factors and performed an in vitro ufmylation assay. Consistent with our prediction, both purified histone H4 protein and histone H4 in the recombinant nucleosomes were mono-ufmylated by UFL1 in vitro (Fig. 4d and Supplementary Figure 3b-c). However, histone H3, H2A, or H2B could not be modified by UFL1 in vitro (Supplementary Figure 3b). Histone H4 contains 11 lysine residues. To pin down the histone H4 ufmylation site, we individually mutated each lysine residue to arginine. We found that mutation of K31 totally abolished UFM1 signal, suggesting that K31 was the only ufmylation site (Fig. 4e). We further confirmed that mutation of lysine at 31 to arginine abolished the ufmylation of H4 in vitro (Supplementary Figure 3d). To further confirm if K31 is important for ATM activation, we transfected wildtype (WT) histone H4 and the K31R mutant constructs into cells and both WT and K31R H4 were incorporated into chromatin (Supplementary Figure 3e, f). Notably, histone H4 K31R expression inhibited ATM signaling and HR, although to a lesser extent than UFL1 knockdown (Fig. 4f, and Supplementary Figure 3g, h). The expression of histone H4K31R did not affect cell cycle profiles (Supplementary Figure 3i). Furthermore, cells expressing H4K31R exhibited defective G2/M checkpoint and increased sensitivity to IR and expression of H4 K31R in ATM depleted cells did not further enhance the sensitivity to IR and G2/M checkpoint (Fig. 4g and Supplementary Figure 3j, k). Collectively, these findings suggest that UFL1 preferentially ufmylates histone H4 at K31, which is important for ATM activation and proper DDR. Histone H4 lysine 31 is also reported to be monoubiquitinated by Cul4A E3 complex which then potentiates gene transcription^37^. To exclude the possibility that  monoubiquitination of H4 lysine 31 also affects ATM activation, we depleted Cul4A, Cul4B, or DDB1 in cells. Depletion of Cul4A, Cul4B, Cul4A+Cul4B, or DDB1 did not affect ATM autophosphorylation (Supplementary Figure 3l–o) and expression of H4K31R did not significantly affect the expression of DNA damage signaling genes (RNA-seq Data, Supplementary Figure 3p, q), suggesting that ATM activation is independent of changes in histone H4 K31 monoubiquitination or gene expression.

### Ufmylation of H4 activates ATM signaling

**Fig. 5:**
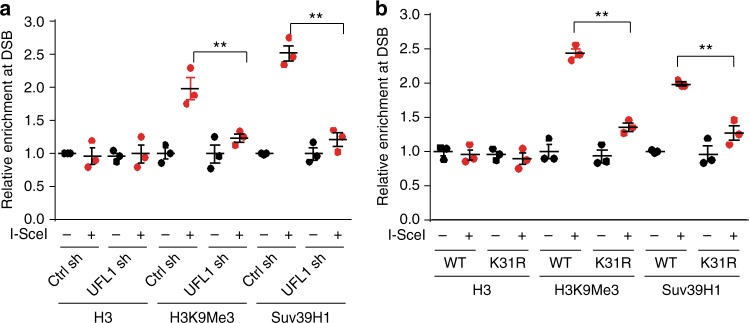
Ufmylation of histone H4 enhances trimethylation of H3K9 at DSB. **a**, **b** Analysis of H3K9 trimethylation and Suv39H1 recruitment by Chromatin IP (ChIP) at DSB sites from MDA-MB-231 ROS8 cells with indicated treatments. The *y*-axis represents relative enrichment of target protein binding DNA compared to input. Target sequence of UFL1 shRNA (not inducible shRNA) in **a** is the same as inducible sh2. The data presented are mean ± s.e.m. for *n* = 3 independent experiments. **p < 0.01. Statistical significance was calculated using one-way ANOVA. Source data are provided as a Source Data file

We next studied how UFL1-mediated histone H4 ufmylation affected ATM activation. We found that depletion of UFL1 led to less DNA-damage induced Tip60 loading to chromatin but did not affect tyrosine phosphorylation of Tip60 (Fig. 4h and Supplementary Figure 3r). We observed a similar result in H4K31R expressing cells (Supplementary Figure 3t). H4K31R expression did not further enhance the sensitivity to IR treatment in Tip60-depleted cells (Supplementary Figure 3s). These results implied that UFL1 regulates ATM activation through Tip60. As Tip60 recruitment to DSBs requires histone H3K9me3, we also examined whether UFL1 regulates H3K9me3. We used a system in which an I-SceI cut site was inserted into a copy of E-cad promoter, and expression of exogenous I-SceI introduced a single DSB in the genome^38^. Following I-SceI induced DSB, we carried out ChIP assays by using antibodies against Suv39h1, H3K9me3, and H3 and followed by qPCR to determine the relative abundance of these modifications at the induced break sites. Consistent with previous results^17^, we observed apparent increase of Suv39h1 and H3K9me3 around DSBs (Fig. 5a, b). In addition, UFL1 depletion inhibited the accumulation of Suv39h1 and H3K9me3 around DSBs (Fig. 5a). Furthermore, we found that overexpression of histone H4K31R also resulted in decreased Suv39h1 and H3K9me3 around DSBs (Fig. 5b). These results suggest that UFL1-mediated histone H4 ufmylation is important for the recruitment of Suv39h1, histone H3K9 methylation at DSB sites, and subsequent Tip60 recruitment and ATM activation.

### Enhancement of UFL1 activity by ATM

**Fig. 6:**
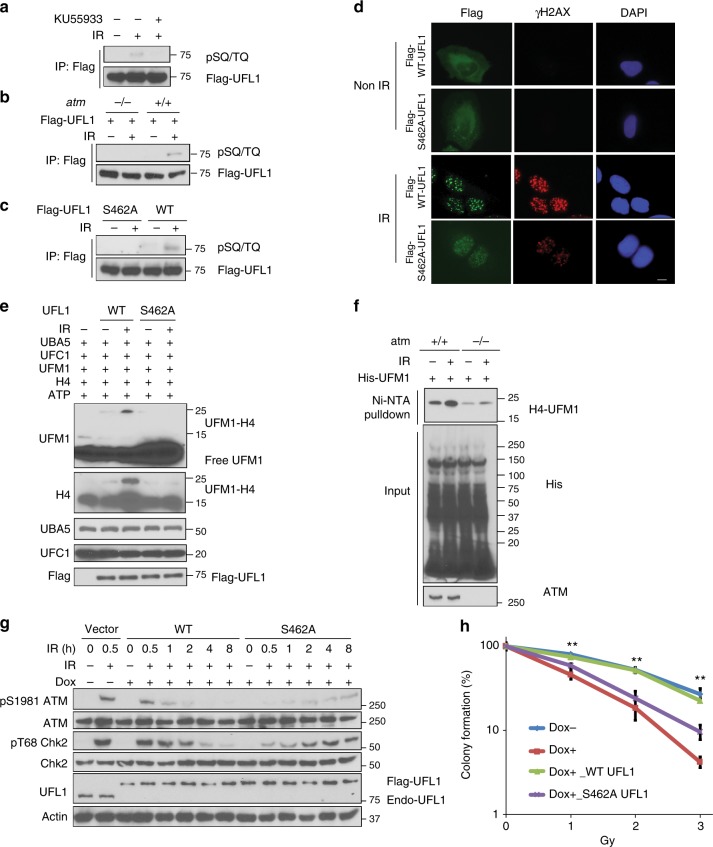
ATM phosphorylates UFL1 and enhances its activity. **a** Flag-UFL1 was expressed in U2OS cells. Following vehicle or ATM inhibitor KU55933 treatment, the cells were irradiated with IR. Flag-UFL1 was immunoprecipitated and blots were probed with phospho-SQ/TQ antibody and Flag antibody. **b** Flag-UFL1 was expressed in *atm+/+*or *atm−/−* MEF cells. Cells were irradiated with IR and UFL1 phosphorylation was then examined as in **a**. **c** Flag-UFL1 or the S462A mutant was expressed in U2OS cells. Cells were treated with or without 10 Gy IR. Thirty minutes later, cells were lysed and incubated with Flag antibody-conjugated agarose beads. The immunoprecipitates were blotted with phospho-SQ/TQ antibody and Flag antibody. **d** Flag-tagged wildtype (WT) and S462A mutant UFL1 expressing cells were treated with or without 2 Gy IR and fixed and stained with indicated antibodies. Scale bars, 10 µm. **e** Flag-tagged UFL1 or S462A mutant expressing U2OS cells were irradiated with 10 Gy IR. Thirty minutes later, UFL1 WT and the S462A mutant were purified from the cells, and incubated with purified UBA5, UFC1, UFL1, UFM1, and H4 protein at 30 ^°^C for 90 min. The reaction was assessed by probing the blot with the indicated antibody. **f** His-UFM1 was expressed in ATM proficient or deficient cells. His-UFM1 conjugated protein was purified under denaturing conditions. The purified samples were blotted with indicated antibodies. **g** Flag-tagged UFL1 or the S462A mutant was expressed in U2OS cells. Cells were harvested at indicated time points following IR and blotted with indicated antibodies. **h** Colony formation assay following IR was performed with UFL1-depleted cells reconstituted with WT UFL1 or the S462A mutant. The data is presented as mean±s.e.m. of *n* = 3 independent experiments. Dots depict individual data points. Statistical significance was calculated using two-way ANOVA. **P < 0.01. Source data are provided as a Source Data file

Increased histone H4 ufmylation following DNA damage suggests that UFL1 activity might be regulated, however, the mechanism by which UFL1 activity is regulated following DNA damage is still unknown. Interestingly, we found that UFL1 is phosphorylated at SQ/TQ motifs in an ATM-dependent manner (Fig. 6a, b), when we utilized an antibody against phospho-SQ/TQ motifs^39^. Inhibition of ATM also impaired the recruitment of UFL1 to DSBs (Supplementary Figure 4a). These results suggest that UFL1 might be a substrate of ATM. We further determined which sites of UFL1 are potentially phosphorylated by ATM. Among potential SQ/TQ sites, we found that mutation of serine 462 to alanine abolished pSQ/TQ signals (Fig. 6c), suggesting that UFL1 S462 is a major ATM phosphorylation site. Because the interaction between NBS1 and UFL1 requires the FHA and BRCT1/2 domains of NBS1, which recognize phospho-residues, we also tested whether this phosphorylation was important for interaction between NBS1 and UFL1. We found that pS462 peptides could pull down NBS1 from cell and interact with the FHA+BRCT1/2 domains of NBS1 in vitro (Supplementary Figure 4b, c**)**. Mutation at S462 (S462A) greatly compromised the recruitment of UFL1 to DSBs (Fig. 6d, Supplementary Figure 4d). These results suggest that the phosphorylation of UFL1 by ATM is important for its interaction with NBS1 and its foci formation. Therefore, UFL1 and ATM form a positive feedback loop. Considering that phosphorylation could also change enzymatic activity, we further tested whether UFL1 activity is changed by this phosphorylation. We found that indeed, purified WT UFL1 from cell lysate displayed increased E3 ligase activity following DNA damage, while the S462A mutant did not (Fig. 6e). Although NBS1 binds to phosphorylated UFL1, it does not affect UFL1 activity (Supplementary Figure 4e), suggesting increased UFL1 activity after DNA damage is not due to NBS1 binding. Therefore, S462 phosphorylation is important for both UFL1 recruitment and activation following DNA damage. To further confirm this, we examined ufmylated histone H4 in control and ATM knockout MEF cells. We found that histone H4 ufmylation was increased in WT MEFs, but no change of histone H4 ufmylation was observed in ATM knockout cells (Fig. 6f), supporting that the UFL1 ufmylation activity is dependent on intact ATM protein. In agreement, ATM activation and Chk2 phosphorylation were compromised and delayed in UFL1-deficient cells reconstituted with the UFL1 S462A and cells expressing the S462A were more sensitive to IR treatment (Fig. 6g, h and Supplementary Figure 4f). These studies strongly suggest that UFL1 and ATM form a positive feedback loop and UFM1 signaling is important for amplification of ATM signaling.

## Disscussion

In spite of many years of extensive studies, how ATM is exactly activated following DSB still remains unclear. The MRN complex functions as a sensor of DNA DSB and is important for ATM activation^6^. The Mre11–RAD50 complex forms a heterotetramer and binds the broken ends of a DSB^40^. NBS1 works as an adapter for interaction between the MRN complex and ATM through a conserved motif at the C-terminus^41–43^. Without any stresses, ATM is inactive and exists in the dimeric or multimeric form; following DNA damage, MRN complex promotes ATM monomerization, and initiates ATM activation process^44^. In addition, ATM activation is also dependent on the acetyltransferase Tip60, which boosts ATM autophosphorylation and activation^16,17^.

Here we show another layer of regulation of ATM activation by connecting the MRN complex and H3K9me3-Tip60 in ATM activation. It has been reported that a protein complex including Kap-1, HP1, and Suv39h1 is recruited to DSBs, and then induces H3K9 trimethylation, and H3K9me3 further spreads over several kilo bases away from DSBs to form a temporary repressive heterochromatin domain^17^. Tip60 binds to H3K9me3 and this binding enhances its acetyltransferase activity; Tip60 then acetylates and activates ATM at DSB^16^. C-Abl phosphorylates Tip60 at Tyr 44, enhances Tip60 binding to H3K9me3, and induces Tip60 binding to ATM to facilitate ATM activation^18,45^. However, how Suv39h1 complex is recruited remains unclear. We show that histone H4 ufmylation is important for the recruitment of Suv39H1 and subsequent H3K9me3 at DSBs and Tip60 recruitment, suggesting that histone H4 ufmylation is important for Suv39H1. It is possible that a reader for H4K31 ufmylation is involved in the recruitment of Suv39H1. In the future, further work is needed to study how histone H4 ufmylation helps Suv39H1 recruitment.

Some studies reported global transient decrease of H3K9me3 following 2 Gy IR, suggesting H3K9me3 is a barrier to DSB repair^46^. However, other studies found H3K9me3 is globally unchanged in insulted cells^16,47^ In addition, it was reported that elevated level of H3K9me3 adjacent to DSB sites is required for recruitment of Tip60 and ATM activation^16,17^. Some recent studies further support the model that H3K9me3 is important for ATM activation. In human stem cells, suppression of H3K9ac and increase of H3K9me3 are detected in the DNA damage sites and contribute to ATM activation and tolerance to IR^48^. Studies in Hutchinson-Gilford Progeria Syndrome show that loss of H3K9me3 correlates with impaired ATM activation^49^. In our study, we also observed local increase of H3K9me3 around the DSB that is dependent on UFL1 and H4K31 ufmylation. Indeed, further studies are required to resolve this discrepancy.

The change of chromatin context is critical for ATM activation and evidences are emerging to demonstrate its importance. Previously, it has been shown that change of the chromatin structure alone could activate ATM^44^. Similarly, histone modification has been shown to regulate ATM activity. In addition to H3K9 trimethylation mentioned above, H3K36 trimethylation and H4K16 acetylation are all suggested to regulate ATM activity. SETD2 mediated trimethylation of H3K36 influences ATM activation and duration^50^. MOF acetylates H4K16 and contributes to ATM autophosphorylation and activation^51^. All these exciting findings support an important role of histone modification in the regulation of ATM activity. Our report that ufmylation of histone H4 is important for ATM activation provides one more layer of regulatory mechanism in this theme. Our results suggest histone H4 ufmylation regulates ATM at least partly through affecting H3K9 methylation; however, we cannot exclude the possibility that histone H4 ufmylation also changes local chromatin structure and affects ATM activation.

Ubiquitination plays an important role in the DDR. Interestingly, ubiquitination also has been reported to enhance ATM activation. Wu et al. found that the E3 ligase Skp2 interacts with NBS1 and induces K63 linked ubiquitination of NBS1 following DNA damage^19^. This modification enhances the interaction between ATM and NBS1, and assists ATM recruitment to DSB and enhances ATM activation. Previous findings show that E3 ligase RNF8 and RNF168 ubiquitinate their downstream targets and is important for the recruitment of DDR factors such as 53BP1^52–56^. The ubiquitination and ufmylation identified in our study potentially collaborate with each other, promote ATM activation and DNA repair and help the cells survive through the DSB. In the future, the potential crosstalk between ubiquitination and ufmylation signaling will be further investigated.

**Fig. 7:**
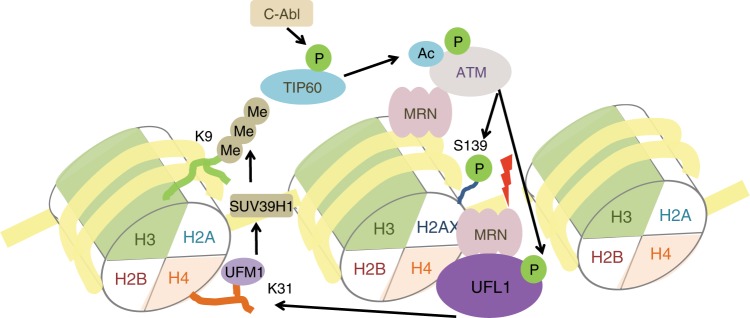
Model for ATM activation by UFL1. When DNA damage occurs, UFL1 is recruited by MRN complex and monoufmylates histone H4, thus recruiting SUV39H1 to DSBs to trimethylate H3K9 thereby forming H3K9me3. Tip60 binds to H3K9me3, acetylates ATM, and promotes ATM activation. C-Abl also phosphorylates Tip60 and enhances Tip60 acetyltransferase activity. Activated ATM phosphorylates UFL1 at Ser462, and enhances its activity thereby amplifying ATM activation signal and forming a positive feedback loop

In summary, here we identify a mechanism for ATM activation and the early DDR signaling. We find ufmylation modification is crucial for DDR and DNA repair. Our study indicates that UFL1 functions as an ATM signaling regulator. As a UFM1 E3 ligase, UFL1 is recruited to chromatin via the MRN complex, and modifies histone H4 at K31. This ufmylation enhances recruitment of Suv39h1 to DSBs, histone H3 lysine 9 trimethylation, Tip60 recruitment, and subsequent ATM activation. UFL1 itself is phosphorylated at S462 by ATM, which is important for its recruitment and boosts its E3 ligase activity, forming a positive feedback loop to amplify ATM activation (Fig. 7). These findings might open up a direction in the study of early DDR signaling.

## Methods

### Cell culture, plasmids, and reagents

U2OS (ATCC), U2OS (TA-induced system) (Kindly provided by Dr. Xiaochun Yu), and HEK 293T (ATCC) cells were cultured in DMEM supplemented with 10% FBS. NBST cells were cultured in DMEM supplemented with 15% FBS. MDA-MB-231 ROS8 cells (Kindly provided by Dr. Stephen Baylin, Johns Hopkins University) were cultured in DMEM supplemented with 10% FBS and Zeocin and Blasticidin treatment. All cell lines were kept in a humidified 37 °C 5% CO_2_ 5%O_2_ incubator.

Flag-His-Ufm1ΔC2, UBA5-GST, UFC1-GST, and UFL1-MBP plasmids were described previously^22^. UFL1-myc^25^ was used as template and UFL1 PCR product were inserted into pIRES vector with Flag-S-SBP tag. UFL1S462A, UFL1 Δ1, Δ2, and Δ3 mutants were generated by site-mutagenesis kit.

Anti-UFL1 antibody (A303-456A, 1:1000 for western blot), anti-NBS1 antibody (A301-284A, 1:1000 for western), anti-DDB1 (A300-462A, 1:1000 for western), and anti-Cul4B (A303-864A, 1:1000 for western) were purchased from Bethyl. Anti-actin (A5316, 1:10,000), anti-UFL1 (HPA030558, 1:1000 for detecting foci), and anti-α tubulin (DM1A, 1:5000 for western and detecting foci) were purchase from Sigma. Anti-ATM (2873, 1:1000 for western), anti-pSer1981 ATM (13050, 1:1000 for western), anti-Mre11 (4847, 1:1000 for western), anti-Rad50 (3427, 1:1000 for western), anti-Suv39h1 (8729, 1:1000), anti-Cul4A (2669, 1:1000 for western), anti-SQ/TQ motif (9607, 1:1000 for western), anti-Chk2 (2662, 1:1000 for western), anti-phosphotyrosine (9411, 1:1000 for western), anti-DNAPK (4602, 1:1000 for western), anti-H3K79me1 (12522, 1:1000 for western), anti-H3K4me1 (4326, 1:1000 for western), anti-H3K4me2 (9325, 1:1000 for western), anti-H2BK120ub (5546, 1:1000 for western), anti-H3K27me2 (9728, 1:1000 for western), anti-H4K20me3 (5737, 1:1000 for western), and anti-phosphoChk2 (2197, 1:1000 for western) antibodies were purchased from Cell Signaling. CTIP antibody (61141, 1:1000 for IF) was purchased from Active Motif. Anti-Tip60 (07-038, 1:1000 for western), anti-53BP1 (mab3802, 1:1000 for western and IF), anti-MDC1 (05-172, 1:1000), anti-Suv39h1 (MABE552, 1:1000 for western), and anti-γH2AX (05-636, 1:1000 for IF) antibodies were purchased from Millipore. Anti-UBA5 (ab177478, 1:1000 for western), anti-UFC1 antibodies (ab189252, 1:1000 for western), anti-H2A (ab18255, 1:1000 for western), anti-H2B (ab1790, 1:1000 for western), anti-H3 (ab1791, 1:3000 for western and 1:1000 for CHIP), anti-H4 (ab10158, 1:1000 for western, and 1:100 for CHIP), anti-H4K16ac (ab109463, 1:100 for CHIP), anti-H3K79me2 (ab3594, 1:1000 for western), and anti-H3K9me3 (ab8898, 1:1000) antibodies were purchased from Abcam. Anti-UFM1 (SC-84652, 1:1000 for IF and western), anti-RPA (sc-56770, 1:1000 for IF), anti-Rad51 (sc-8349, 1:1000 for IF), and anti-BRCA1 (sc-6954, 1:1000 for IF) antibodies were purchased from Santa Cruz. Rabbit 53BP1 antibody (NB100-304, 1:1000) and NBS1 antibody (NB100-143, 1:1000) for foci were purchased from Novus Biologicals. For immunoprecipitation assay, Anti-IgG, Light Chain Specific antibodies were used (Jackson immunoresearch).

Lipofectamine 2000 Transfection Reagent (Invitrogen) and Mirus TransIT Transfection Reagent (Mirus Bio LLC) were used for carrying out transfections following the manufacturer’s protocols.

### Chemicals

Triamcinolone acetonide, Doxycyline and the ATM inhibitor KU55933 (Sigma) were used in this study.

### RNA interference

UFL1 shRNA sh1 (oligo1 GAAACACTTCTGTGTCAGAAA, targeting 3′UTR), (oligo2 GCTCTGGAACATGGGTTGATA, targeting CDS) were inserted into Tet-on PLKO.1 vector. Tet-on PLKO.1 vector. UFM1 shRNA sh1 (oligo1 GTGTTGGAAGTTGTTAATATC, targeting CDS), and sh2 (oligo2, CAATGATGGAATAGGAATAAA targeting CDS) were inserted into Tet-on PLKO.1 vector. Cul4A shRNA sh1 (target sequence GCAGAACTGATCGCAAAGCAT) and sh2 (target sequence GGACAAGAAGATGTTACTAAA) were purchased from Sigma. Cul4B shRNA sh1 (target sequence GCAATTCTTCAGAAAGGTTTA) and sh2 (target sequence GCCATGAAAGAAGCATTTGAA) were purchased from Sigma. DDB1 shRNA1 (target sequence CGACCGTAAGAAGGTGACTTT) and sh2 (target sequence CCTTGATTGGTGTTGCCAGTT) were purchased from Sigma. Lentiviruses were made according to manufacturer’s protocol. Tip60 siRNA was purchased from Santa Cruz.

### Western blot and immunoprecipitation

Cells were lysed with NETN buffer (20 mM Tris-HCl, pH 8.0, 100 mM NaCl, 1 mM EDTA, 0.5% Nonidet P-40 with 50 mM β-glycerophosphate, 10 mM NaF, and 1 mg per ml each of pepstatin A and aprotinin. Lysates were centrifuged at 9600×*g* for 15 min to remove cellular debris. After centrifugation, proteins of interest were immunoprecipitated by incubating lysates with 2 µg of indicated antibodies, and 20 µl protein A or protein G Sepharose beads (Amersham Biosciences) for 2 h or overnight at 4 °C. The immuno-precipitates were washed with ice cold NETN buffer, and centrifuged at 800×*g* for 1 min. This step was repeated twice and 40 µl 1x Laemmli buffer was added to the immuno-precipitates. Samples were boiled, followed by SDS–PAGE separation. Samples in SDS-PAGE gels were then transferred to PVDF membrane with semi-dry method (Trans-Blot® Turbo™ Transfer System, Bio-Rad). The membrane was blocked with 5% milk for 1 h and blotted with indicated antibodies overnight, washed twice with TBST buffer, and blotted with goat anti-rabbit HRP or goat anti-mouse HRP secondary antibodies (Jackson Immunoresearch) for 1 h. After three washes with TBST buffer, western blots were analyzed by Tanon 5200 Image System and Image Analysis software. Uncropped blots and gels are provided in Supplementary Fig. 5.

### Irradiation

Unless mentioned, cells were irradiated with 0.5 Gy for immunofluorescence studies and 2 Gy for western blot/co-immunoprecipitation assays. Cells were analyzed 30 min following irradiation unless noted otherwise.

### Chromatin fractionation assay

Cells were harvested, and washed once with PBS. Then the cells were lysed with NETN buffer with low salt (20 mM Tris-HCl, pH 8.0, 10 mM NaCl, 1.5 mM MgCl_2_, 1 mM EDTA, 0.5% Nonidet P-40, 20 mM NaF, 1 mM Na_3_VO_4_, 1 μg per ml aprotinin, and 1 μg per ml pepstatin) for 20 min. The lysates were centrifuged at 15,000 × *g* for 10 min. The supernatant was aspirated off and the chromatin pellet was washed with PBS and centrifuged at 15,000×*g* for 2 min for three times. The pellet was resuspended in 0.2 M HCl for 30 min on ice. The soluble exact was neutralized with 1 M NaOH for western blot.

### Flag-His-UFM1 purification

Cells stably expressing Ufm1ΔC2-Flag-His were irradiated and then lysed with denaturing buffer with 8 M urea, 50 mM Na_2_HPO_4_ pH 8.0, 0.3 M NaCl, and 1 mM PMSF. After sonication, the supernatant was incubated with nickel bead for 2 h. Following 3 times wash with buffer containing 8 M Urea, 50 mM Na_2_HPO_4_ pH 8.0, 0.5 M NaCl, and 10 mM imidazole, the His-tagged protein was eluted off with 50 mM Tris-Cl (pH8.0), 50 mM NaCl, 300 mM Imidazole, and 0.1 mM EDTA buffer. The purified proteins were dialyzed with Amico ultra-spin column and then incubated with Flag agarose beads for 1 h. The binding protein was eluted with Flag peptide.

### Mass spectrometric analysis

The purification samples (Flag-His only *n* = 1, and Flag-His-UFM1 *n* = 1) were subjected to SDS-PAGE followed by Coomassie staining. Bands were excised from the gel and submitted for mass spectrometric analysis at the Taplin Mass Spectrometry Facility, Harvard Medical School, Boston, MA. Excised gel bands were cut into ~1 mm^3^ pieces. Gel pieces were then subjected to a modified in-gel trypsin digestion procedure. Gel pieces were washed and dehydrated with acetonitrile for 10 min. followed by removal of acetonitrile. Pieces were then completely dried in a speed-vac. Rehydration of the gel pieces was with 50 mM ammonium bicarbonate solution containing 12.5 ng per µl modified sequencing-grade trypsin (Promega, Madison, WI) at 4 °C. After 45 min, the excess trypsin solution was removed and replaced with 50 mM ammonium bicarbonate solution to just cover the gel pieces. Samples were then placed in a 37 °C room overnight. Peptides were later extracted by removing the ammonium bicarbonate solution, followed by one wash with a solution containing 50% acetonitrile and 1% formic acid. The extracts were then dried in a speed-vac (~1 h). The samples were then stored at 4 °C until analysis.

On the day of analysis, the samples were reconstituted in 5–10 µl of HPLC solvent A (2.5% acetonitrile, 0.1% formic acid). A nano-scale reverse-phase HPLC capillary column was created by packing 2.6 µm C18 spherical silica beads into a fused silica capillary (100 µm inner diameter×~30 cm length) with a flame-drawn tip. After equilibrating the column each sample was loaded via a Famos auto sampler (LC Packings, San Francisco CA) onto the column. A gradient was formed and peptides were eluted with increasing concentrations of solvent B (97.5% acetonitrile, 0.1% formic acid). Eluted peptides were subjected to electrospray ionization and then entered into an LTQ Orbitrap Velos Pro ion-trap mass spectrometer (Thermo Fisher Scientific, Waltham, MA). A high resolution scan was done at 60,000 resolution, followed by 20 low-resolution MS/MS scans in the ion-trap. Peptide sequences (and hence protein identity) were determined by matching protein databases (Uniprot, 2017_10) with the acquired fragmentation pattern by the software program, Sequest Version 3.2 (ThermoFisher, San Jose, CA). The database was indexed based on a trypsin digestion, with two missed cleavages. Fixed modification of 57.0214 Da on cysteine (iodoacetamide) and a variable modification of 15.9949 Da on methionine were considered. The MS1 mass tolerance was 50 ppm and the MS2 tolerance was 1.0 Da. The peptide mass range used was 600–6000 Da. All accepted peptides have a cross-correlation (Xcorr) score of at least 0.5. The data is filtered between a 1-2% peptide false discovery rate. The protein FDR is 8.11%.

### Colony formation assay

1000–3000 U2OS cells with indicated treatment were plated in each well of 6-well plates. Sixteen hours later, cells were exposed to ionizing radiation, and incubated for 10–14 days at 37 °C to allow colony formation. Colonies were stained with methylene blue and counted. Results were normalized to plating efficiencies.

### In vitro ufmylation assay

In vitro ufmylation assay was performed following previous reported method^23^. In brief, recombinant GST-UFM1, GST-UBA5, GST-UFC1, and NBS1- GST were expressed in *E. coli*, and recombinant proteins were purified using glutathione-Sepharose 4B and PreScission protease according to the protocol provided by the manufacturer (Amersham Biosciences). Recombinant His-Rad50 was expressed in SF9 cells and the recombinant proteins were purified using nickel beads according to the protocol supplied by the manufacture (Thermo Fisher Scientific). MBP-Ufl1 was expressed in *E. coli*, and these recombinant proteins were purified with amylose resin according to the protocol supplied by the manufacturer (New England BioLabs). Purified Mre11 protein was purchased from Origene. Purified recombinant proteins were dialyzed with buffer containing 50 mM Tris (pH 8.5), 150 mM NaCl, and 1 mM dithiothreitol. The purified proteins UFM1ΔC2, UBA5, UFC1, MBP-Ufl1, and histone H4, H3, H2A, or H2B (NEB) or nucleosome (Active motif)) were added into the reaction buffer containing 5 mM ATP and 10 mM MgCl_2_ and then incubated at 30 °C for 90 min. SDS sample buffer containing 5% β-mercaptoethanol was added into the mixture to stop the reaction.

### Immunofluorescence staining

Cells were seeded on coverslips and then fixed with 3% paraformaldehyde on ice at indicated timepoints after IR treatment, washed with PBS, and permeabilized for 10 min with 0.5% Triton X-100. Cells were blocked with 5% goat serum and then incubated with primary antibody for 1 h at room temperature. After washing with PBS, FITC, Rhodamine or Alexa405 conjugated secondary antibody (Jackson ImmunoResearch) was added and incubated for 30 min at room temperature. After two times PBS wash, cells were counterstained with 4'6-diamidino-2-phenylindole (DAPI). Finally, the coverslips were mounted with glycerin containing paraphenylenediamine. The coverslips were mounted onto glass slides with anti-fade solution and visualized using a Nikon ECLIPSE E800 fluorescence microscope. The foci intensity was quantified with Image J software.

### Cell cycle analysis and G2/M checkpoint

U2OS cells in 10-cm plates with different treatments were harvested and fixed for 90 min with 70% (v per v) pre-cooled ethanol (−20 °C) and harvested by centrifugation (4 °C, 5 min, 1000×*g*). Staining was performed using 69 μM propidium iodide solution in phosphate-buffered saline containing RNaseA (0.6 μg per ml) for 30 min at 37 °C. The cell cycle distribution was calculated from the resultant DNA histograms using FlowJo software. For cell cycle analysis, unstained cells were used as control.

For G2/M checkpoint analysis, fixed cells were harvested and fixed for 90 min with 70% (v per v) pre-cooled ethanol (−20 °C) and harvested by centrifugation (4 °C, 5 min, 1000 g). Then the cells were stained with phospho-H3 antibody (Abcam, 1:1000) first for 1 h. After washes with PBS, the cells were incubated with FITC-labeled secondary antibody (Jackson ImmunoResearch, 1:1000). Then cells were incubated with 69 μM propidium iodide and analyzed by FACS. Negative controls were unstained cell, PI only, phospho-H3 staining only and control IgG only stained cells to set up the gates.

### GST fusion protein purification and pulldown assay

NBS1 or NBS1 FHA+BRCT1/2 was inserted into vector pEX-C-GST, which expressed GST tag at C terminus. NBS full length GST proteins and FHA+BRCT1/2 GST proteins were expressed in BL21 *E. coli*. Bacteria were treated with 0.25 mM isopropyl β-d-thiogalactoside overnight at 18 ^°^C. Cell pellets were resuspended in lysis buffer with protease inhibitor (Roche) and sonicated. The supernatant was incubated for 4 h with glutathione-agarose beads at 4 °C. After washing, GST fusion proteins were eluted with glutathione. Then cell lysates were incubated with 1–2 μg of GST fusion protein and 40 μl of glutathione-agarose beads in a total of 1 ml of NETN buffer at 4 °C on a rotator for 2 h. After the beads had been washed three times with NETN buffer, the protein was eluted for SDS–PAGE gel analysis.

UFL1 phospho peptide (DDSDDEpSQSSHTG) and non-phospho peptide (DDSDDESQSSHTG) were synthesized by Genscript. The peptides were covalently conjugated to KLH beads following the procedure provided by manufacture. Then the peptide conjugated beads were incubated with purified GST fusion protein. After washes, the beads were boiled and analyzed by SDS-PAGE.

### Nucleosome preparation

Nucleosomes were purified with Nucleosome purification kit (Active Motif) with modifications. In brief, U2OS cells with different treatments were lysed with the lysis buffer containing protease inhibitor and phosphatase inhibitor on ice, and centrifuged at 3000 × *g* for 10 min. The lysis was monitored by light microscopy. The nuclei were incubated in NETN buffer plus protease inhibitor and phosphatase inhibitor and centrifuged at 3000 × *g* for 10 min. The pellets were re-suspended with digestion buffer (every 50 µl Chromatin with 2.5 µl Enzyme) and incubated at 37^o^C. The digestion was monitored by agarose gel analysis. The reaction was stopped with ice-cold EDTA on ice for 10 min. The digested nucleosome samples were centrifuged for 10 min at 18,000xg, 4 °C. The supernatants were further separated by 5–20% (w per v) linear sucrose gradients centrifuge to isolate mononucleosome. Purified mononucleosomes were dialyzed with NETN buffer and the next step experiments were performed.

### Inducible single DSB system for monitoring foci formation

U2OS cells stably expressing RFP-I-SceI-GR was used for monitoring single foci formation^35^. The synthetic glucocorticoid (GR) ligand triamcinolone acetonide (TA purchased from Sigma) was added (0.1 µM) into medium to induce the translocation of RFP-I-SceI-GR from cytoplasm into nucleus. Photos were taken using a Nikon eclipse 80i fluorescence microscope.

### Homologous recombination assay

U2OS DR-GFP cells were used for homologous recombination assay^39^. Cells with indicated treatment were transfected with pCBA-I-SceI and pCherry. Two days later, cells were harvested and analyzed by fluorescence-activated flow cytometry (FACS) to examine GFP positive cells. The controls were unstained cells, GFP only cells, and mCherry only cells. After cells were selected in the FSC/SSC dot plot to remove debris, they were gated to exclude cellular aggregates in the FSC/FSC dot plot. Gates of positive cells were set and compared with a control sample (unstained cells) with no detectable fluorochrome expression. Results were normalized to control group.

### NHEJ assay

Linearized pEGFP-Pem1-Ad2 and pCherry were co-transfected into cells with indicated treatment. Forty-eight hours later, cells were harvested and fixed with 2% paraformaldehyde and analyzed by FACS (Calibur instrument, BD Biosciences). For HR or NHEJ reporter assay, the controls were unstained cells, GFP only cells, and mCherry only cells. After cells were selected in the FSC/SSC dot plot to remove debris, they were gated to exclude cellular aggregates in the FSC/FSC dot plot. Gates of positive cells were set and compared with a control sample (unstained cells) with no detectable fluorochrome expression.

### Chromatin immunoprecipitation assay

MDA-MB-231ROS8 cells were used for ChIP assay. One day after transfection of I-SceI, about 5×10^7^ cells were treated with 1% formaldehyde for 10 min at room temperature to crosslink proteins to DNA. Glycine was added and incubated at room temperature for 5 min to stop the cross-linking. Cells were harvested and the pellets were resuspended in cell lysis buffer [5 mM Pipes (KOH), pH 8.0, 85 mM KCl, 0.5% NP-40] containing the following protease inhibitors; 1 μg per ml leupeptin, 1 μg per ml aprotinin, and 1 mM PMSF and incubated for 10 min on ice. Nuclei were pelleted by centrifugation (6000 × *g* for 5 mins). Nuclei were then resuspended in nuclear lysis buffer [50 mM Tris, pH 8.1, 10 mM EDTA, 1% SDS containing the same protease inhibitors as in cell lysis buffer] and sonicated to shear chromatin to an average size of 0.6 kb. Once centrifuged until clear, the lysates were precleared overnight with salmon sperm DNA/protein-A agarose slurry. 20% of each supernatant was used as input control and processed with the cross-linking reversal step. The rest of the supernatant (about 80% of the total) was incubated with 5 μg of the indicated antibody overnight at 4 °C with rotation. Complexes were washed four times, once in high salt buffer (50 mM Tris–Cl, pH 8.0, 500 mM NaCl, 0.1% SDS, 0.5% deoxycholate, 1% NP-40, 1 mM EDTA), once in LiCl buffer (50 mM Tris–Cl, pH 8.0, 250 mM LiCl, 1% NP-40, 0.5% deoxycholate, 1 mM EDTA), and twice in TE buffer (10 mM Tris–Cl, pH 8.0, 1 mM EDTA, pH 8.0). Beads were resuspended in TE containing 50 mg per ml of RNase and incubated for 30 min. Beads were washed with water and elution buffer (1% SDS, 0.1 M NaHCO_3_) was added for 15 min. Crosslinks were reversed by adding 10 μg per ml RNase and 5 M NaCl to a final concentration of 0.3 M to the eluents and incubated in a 65 °C water bath for 4–5 h. Two volumes of 100% ethanol were added to precipitate overnight at –20 °C. DNA was pelleted and resuspended in 100 μl of water, 2 μl of 0.5 M EDTA, 4 μl 1 M Tris, pH 6.5, and 1 μl of 20 mg per ml Proteinase K was added and incubated for 1–2 h at 45 °C. DNA was then purified and used in PCR reactions. The PCR primers for ChIP, close to the I-SceI cut site, were as follows: Forward: 5′-CCCTCTCAGTGGCGTCGGAACT-3′ Reverse: 5′- CCCACCCTCTGATGAGTACCT-3′. Amplification was performed using the following program: 95 °C for 5 min, 1 cycle; 95 °C for 45 s, 56 °C for 30 s, and 72 °C for 30 s, 30 cycles; 72 °C for 10 min, 1 cycle. As an internal control for the normalization of the specific fragments amplified, a locus outside the region of the DSB was amplified, in this case FKBP5, using the input control sample as template. The internal control (FKBP5) primers were as follows: Forward: 5′-CAGTCAAGCAATGGAAGAAG-3′;

Reverse: 5′-CCCGTGCCACCCCTCAGTGA-3′ After Q-PCR amplification, the FKBP5 input controls for untransfected (no DSB) and I-SceI transfected (DSB) cells were used to normalize the untransfected and transfected samples respectively. After normalization, the relative levels of the indicated proteins on a DSB were calculated using the formula: [IP I Sce-1/Input ISce-1]/ [IP untransfected /Input untransfected]. All Q-PCR reactions were performed in triplicate, with the SEM values calculated from three independent experiments.

### RNA-seq analysis

Cells were washed twice with cold PBS and harvested using 350 μl of lysis buffer. Total RNA was extracted following the manufacturer’s instructions (RNeasy kit, Qiagen). The extracted RNA was quantified using a NanoDrop ND-1000 Spectrophotometer. For the library preparation, the Illumina TruSeq Stranded mRNA Library Prep Kit High Throughput was used according to the manufacturer’s instructions and two lanes of 50 bp single-end reads were run on HiSeq 4000. Reads were aligned to the human genome version GRCh37.75 using TopHat v2.1.066. Read counts were obtained using feature Counts function in Subread v1.5.267 and read counts were normalized and tested for differential gene expression using the DESeq2 workflow. Multiple testing correction was applied using the Benjamini–Hochberg method.

### RNA-seq data analysis and pathway enrichment test

The raw sequence reads were aligned to human genome hg38 with the STAR aligner (v2.3.0e). GENCODE gene annotation model GRCh38.p10 release 27 was used to guide the STAR alignment. Then the gene level expression was quantified by FeatureCounts (v1.4.4). The gene level read counts data was normalized using the trimmed mean of M-values normalization (TMM) method to adjust for sequencing library size difference. The normalized data was further loaded to the voom function of the R/Bioconductor package limma for estimating the mean and variance relationship. Subsequently, differential expression between KR and WT was computed by moderated t-test implemented in limma. Genes with Benjamini–Hochberg (BH) FDR corrected *P* value ≤ 0.05 and fold change ≥1.2 were considered significant. Canonical functional pathways enriched in the differentially expressed genes (DEGs) were identified through the use of Ingenuity Pathway Analysis (IPA) (QIAGEN Inc., https://www.qiagenbioinformatics.com/products/ingenuity-pathway-analysi). Again, BH’s FDR approach was used to correct for multiple tests by IPA.

### Statistical analysis

Data in bar and line graphs are presented as mean±SD or mean±s.e.m. of at least three independent experiments. Comparisons were carried out with a two-tailed unpaired Student’s *t*-test and two-way or one-way ANOVA using graph pad prism (^∗^*p* < 0.05, ^∗∗^*p* < 0.01).

### Reporting summary

Further information on experimental design is available in the Nature Research Reporting Summary linked to this article.

## Supplementary information

Supplementary information

Description of Additional Supplementary Files

Supplementary Data 1

Reporting Summary

Source Data

## Data Availability

RNA-seq data used for Supplementary Figure 3p, q have been deposited to the GEO database under the accession number GSE126451. The mass spectrometry data have been deposited to ProteomeXchange via the PRIDE partner repository with the data set identifier PXD012729. The source data underlying Figs. 1a, e, f, g, i, 2a, c, d, 3, 4b–h, 5, 6a–c, e–h and Supplementary Figs 1, 2a–j, l–o, 3, 4b–f are provided as a Source Data file. A reporting summary for this Article is available as a Supplementary Information file. All data supporting the findings of this study are available from the corresponding author on reasonable request.
