# Supplementary material for: UFL1 promotes histone H4 ufmylation and ATM activation
Source: Nat Commun. 2019 Mar 18;10:1242. doi: 10.1038/s41467-019-09175-0 (PMC6423285; doi:10.1038/s41467-019-09175-0)
Supplement: Supplementary file 1 — Supplementary information [file 41467_2019_9175_MOESM1_ESM.pdf]

UFL1 promotes histone H4 ufmylation and ATM activation

Bo Qin[1, 2,7], Jia Yu[2,7], Somaira Nowsheen [1,3], Minghui Wang[4], Xinyi Tu[1], Tongzheng Liu[5], Honglin Li[6], Liewei Wang[2], Zhenkun Lou[1*]

[1]Division of Oncology, Mayo Clinic, Rochester, MN, USA

[2]Division of Clinical Pharmacology, Department of Molecular Pharmacology and Experimental Therapeutics, Mayo Clinic, Rochester, MN, USA

[3]Mayo Medical Scientist Training Program, Mayo Medical School and Mayo Graduate School, Mayo Clinic, Rochester, MN, USA

[4]Department of Genetics and Genomic Sciences, Icahn Institute of Genomics and Multiscale Biology, Icahn School of Medicine at Mount Sinai, 1470 Madison Avenue, New York, NY,USA

[5]Institute of Tumor Pharmacology, Jinan University, Guangzhou, China

[6]Department of Biochemistry & Molecular Biology, Cancer Center, Georgia Regents University, Augusta, GA, USA

[7]Both authors contributed equally to this work.

* Correspondence should be addressed to Z.L.: lou.zhenkun@mayo.edu

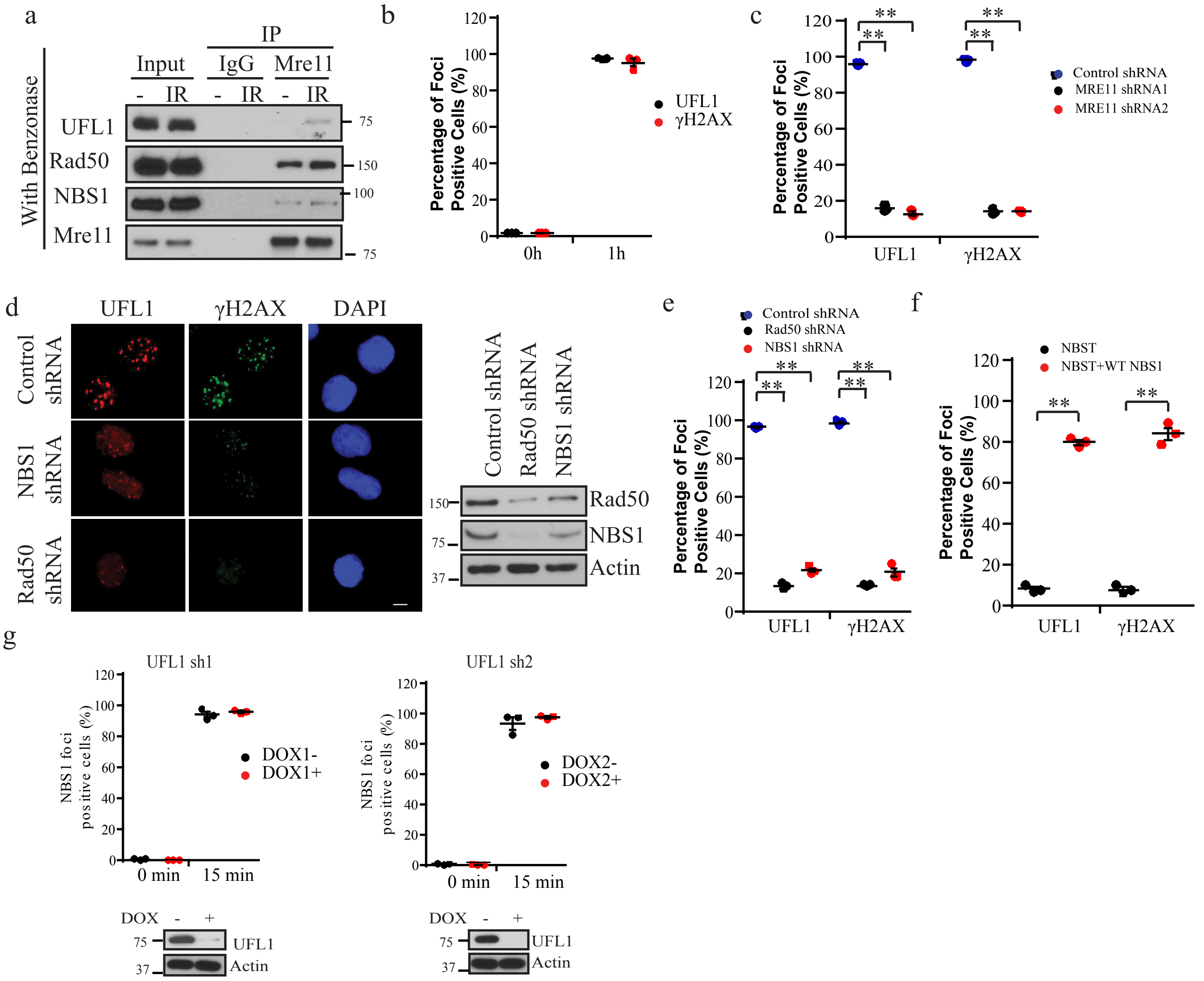

Supplementary Fig.1 UFL1 is recruited to DSBs by the MRN complex.

(**a**) U2OS cells were treated with or without 2 Gy IR. Mre11 was immunoprecipitated from lysates with Benzonase treatment and the immunoprecipitates were analyzed with indicated antibodies.
(**b**) Quantification of UFL1 and γH2AX foci positive cells. The data presented are mean ± SD for n=3 independent experiments. Related to **Fig.1b**. (**c**) Quantification of UFL1 and γH2AX foci positive cells in cells transfected with control shRNA and two different Mre11 shRNAs. The data presented are mean ± SEM for n=3 independent experiments. ** indicates p<0.01. Statistical significance was calculated using 1-way ANOVA test. Related to **Fig.1e**. (**d**) Immunofluorescence of UFL1 and γH2AX in NBS1 or Rad50 depleted U2OS cells after 0.5 Gy IR. Scale bars, 10 µm. (**e**) Quantification of UFL1 and γH2AX foci positive cells. The data presented are mean ± s.e.m. for n=3 independent experiments. ** indicates p<0.01. Statistical significance was calculated using 1-way ANOVA test. Related to **Supplementary Fig.1d**. (**f**) Quantification of UFL1 and γH2AX foci positive cells in NBST and NBST+ wild-type (WT) NBS1 cells in **Fig.1f**. The data presented are mean ± s.e.m. for n=3 independent experiments. ** indicates p<0.01. Statistical significance was calculated using 2-way ANOVA. (**g**) Quantification of NBS1 foci formation in UFL1 knockdown cells (The data presented are mean ± SD for n=3 independent experiments.

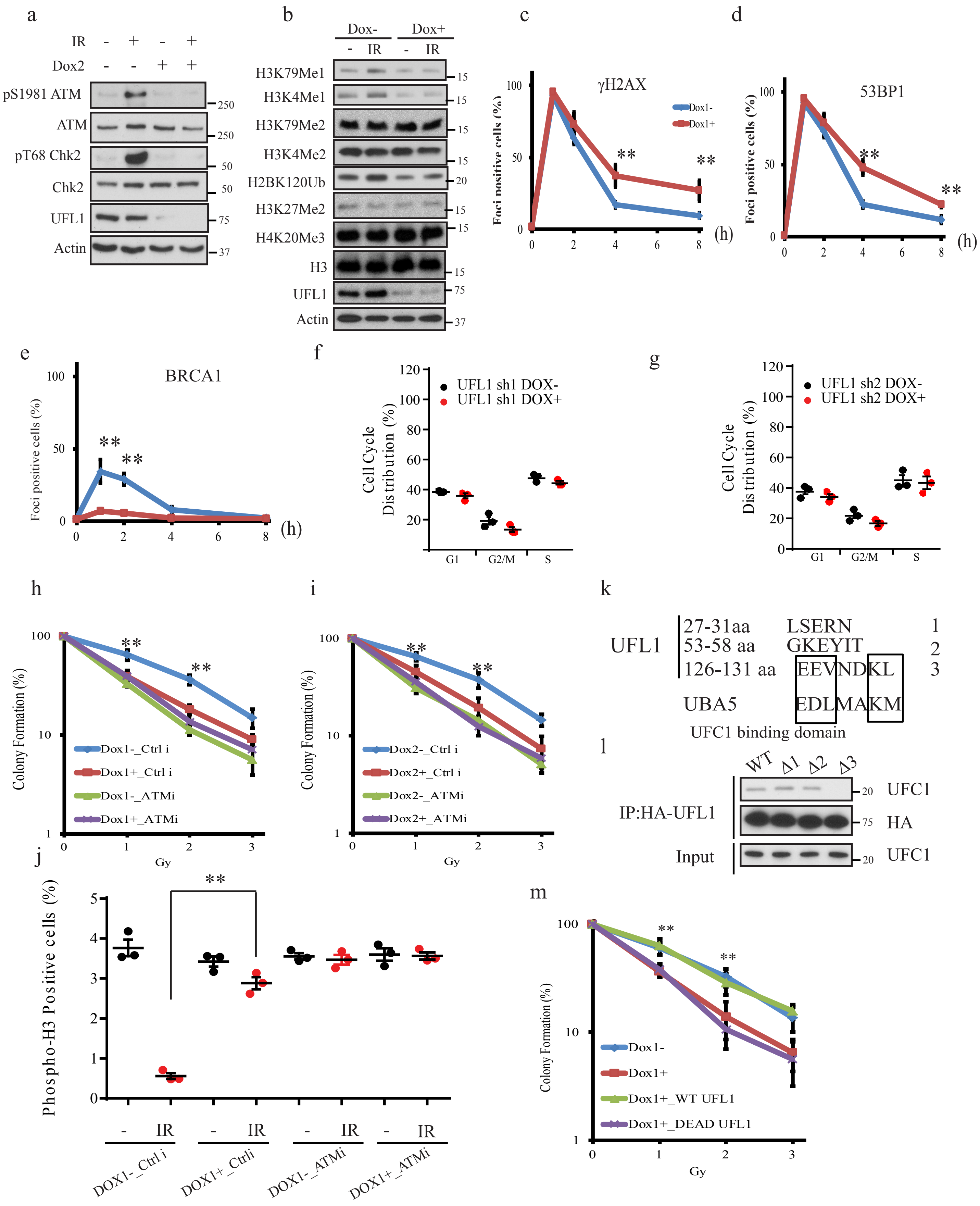

a
IR - + - +
Dox2 - - + +
pS1981 ATM
ATM
pT68 Chk2
Chk2
UFL1
Actin
250
250
50
50
75
37
b
Dox- Dox+
- IR - IR
H3K79Me1
H3K4Me1
H3K79Me2
H3K4Me2
H2BK120Ub
H3K27Me2
H4K20Me3
H3
UFL1
Actin
15
15
15
15
20
15
15
15
75
37
c
γH2AX
Dox1-
Dox1+
Foci positive cells (%)
100
50
0
0 2 4 6 8 (h)
**
**
d
53BP1
Foci positive cells (%)
100
50
0
0 2 4 6 8 (h)
**
**
e
BRCA1
Foci positive cells (%)
100
50
0
0 2 4 6 8 (h)
**
**
f
UFL1 sh1 DOX-
UFL1 sh1 DOX+
Cell Cycle Dis trib ution (%)
120
100
80
60
40
20
G1 G2/M S
g
UFL1 sh2 DOX-
UFL1 sh2 DOX+
Cell Cycle Dis trib ution (%)
120
100
80
60
40
20
0
G1 G2/M S
h
Colony Formation (%)
100
10
1
0 1 2 3
Gy
**
**
Dox1-_Ctrl i
Dox1+_Ctrl i
Dox1-_ATMi
Dox1+_ATMi
i
Colony Formation (%)
100
10
1
0 1 2 3
Gy
**
**
Dox2-_Ctrl i
Dox2+_Ctrl i
Dox2-_ATMi
Dox2+_ATMi
k
UFL1
27-31aa LSERN 1
53-58 aa GKEYIT 2
126-131 aa EEVNDKL 3
UBA5 EDLMAKM
UFC1 binding domain
l
WT Δ1 Δ2 Δ3
IP:HA-UFL1
Input
20 UFC1
75 HA
20 UFC1
j
Phospho-H3 Positive cells (%)
5
4
3
2
1
0
**
- IR - IR - IR - IR
DOX1-_Ctrl i
DOX1+_Ctrli
DOX1-_ATMi
DOX1+_ATMi
m
Colony Formation (%)
100
10
1
0 1 2 3
Gy
**
**
Dox1-
Dox1+
Dox1+_WT UFL1
Dox1+_DEAD UFL1

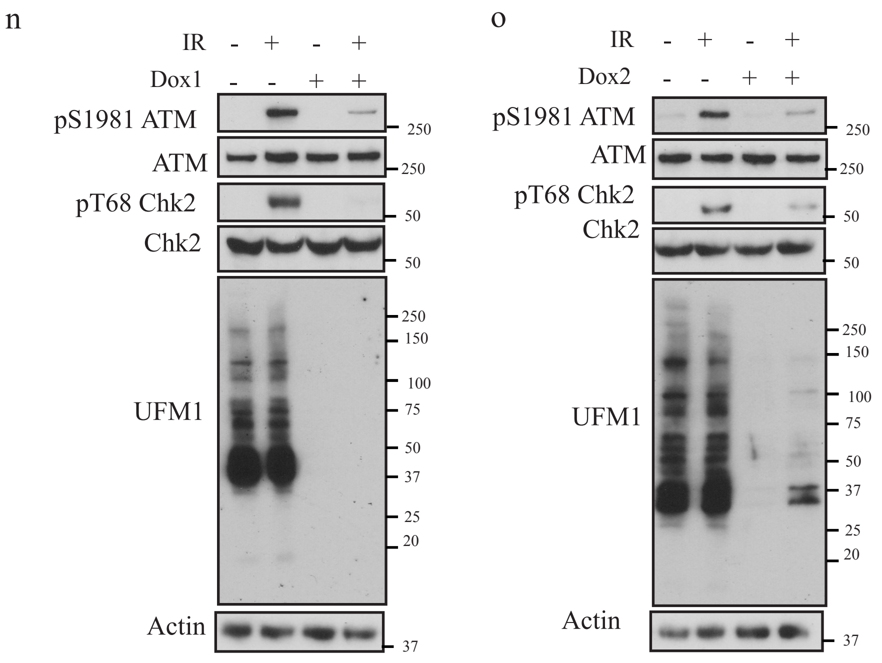

Supplementary Fig.2. UFL1 regulates ATM signaling.

(**a**) U2OS cells stably expressing UFL1 Tet-on shRNA sh2 treated with or without doxycycline and exposed to ±2Gy IR. 30 minutes later, cells were lysed and analyzed with indicated antibodies. (**b**) U2OS cells stably expressing UFL1 Tet-on shRNA sh1 were treated with or without doxycycline and exposed to ±2Gy IR. 30 minutes later, cells were lysed and analyzed with indicated antibodies. (**c-e**) Quantification of γH2AX, 53BP1, and BRCA1 foci positive cells in control (Dox-) and UFL1 knockdown (Dox+) U2OS cells. The data presented are mean ± s.e.m. for n=3 independent experiments. ** indicates $p<0.01$. Statistical significance was calculated using 2-way ANOVA.. (**f-g**) U2OS cells stably expressing UFL1 Tet-on shRNA sh1 and sh2 were treated with or without doxycycline. Cell cycle distribution was analyzed by FACS. The data presented are mean ± s.e.m. for n=3 independent experiments. (**h-i**) Colony formation of U2OS cells stably expressing UFL1 Tet-on shRNA sh1 (**h**) and sh2 (**i**) were treated with or without doxycycline/ ATM siRNA. The data presented are mean ± s.e.m. for n=3 independent experiments. ** indicates $p<0.01$. Statistical significance was calculated using 2-way ANOVA. (**j**) Analysis of phospho-H3 positivity in U2OS cells stably expressing UFL1 Tet-on shRNA sh1 and treated with or without doxycycline/ ATM siRNA. The data presented are mean ± s.e.m. for n=3 independent experiments. ** indicates $p<0.01$. (**k**) Alignment of UFL1 conserved region in 1-212 aa (previously reported UFC1 binding region) with UBA5 UFC1 binding domain. (**l**) U2OS cell expressing wildtype (WT) and mutations of conserved region listed in **k** were lysed and immunoprecipitated with HA-conjugated agarose beads. The immunoprecipitates were blotted with indicated antibodies. (**m**) Colony formation of U2OS cells stably expressing UFL1 Tet-on shRNA sh1 and treated with or without doxycycline and reconstituted with wildtype (WT) or ligase dead (DEAD) UFL1. The data presented are mean ± s.e.m. for n=3 independent experiments. ** indicates $p<0.01$. Statistical significance was calculated using 2-way ANOVA. (**n-o**) U2OS cells were stably infected with lentiviruses encoding two different Tet-on shRNAs for UFM1. After induction, the cells were treated with or without 2 Gy IR. 30 minutes later, cells were lysed and blotted with indicated antibodies.

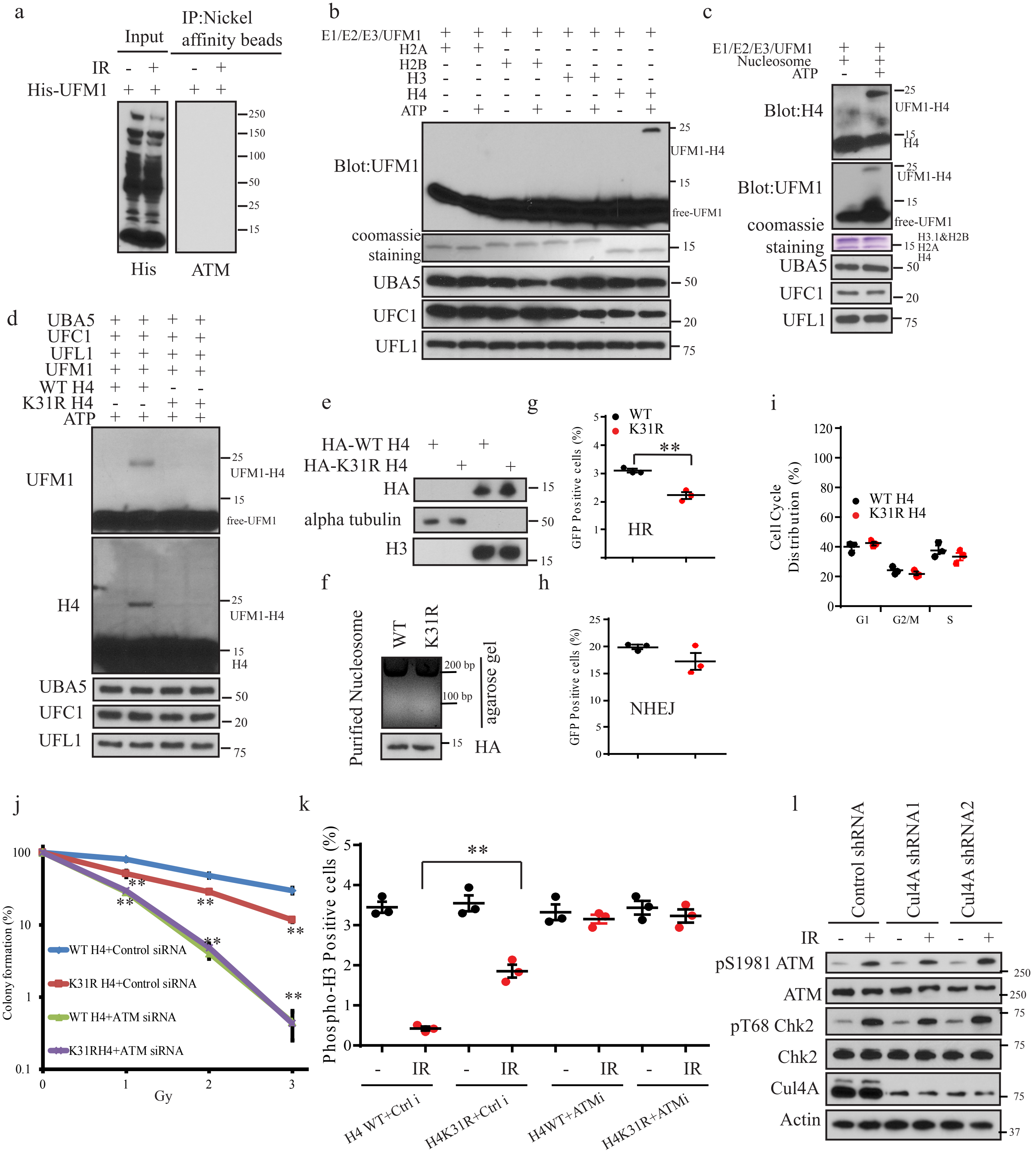
a
Input
IP:Nickel affinity beads
IR
His-UFM1
His
ATM
b
E1/E2/E3/UFM1
H2A
H2B
H3
H4
ATP
Blot:UFM1
UFM1-H4
free-UFM1
coomassie staining
UBA5
UFC1
UFL1
c
E1/E2/E3/UFM1
Nucleosome
ATP
Blot:H4
UFM1-H4
H4
Blot:UFM1
UFM1-H4
free-UFM1
coomassie staining
H3.1&H2B
H2A
H4
UBA5
UFC1
UFL1
d
UBA5
UFC1
UFL1
UFM1
WT H4
K31R H4
ATP
UFM1
UFM1-H4
free-UFM1
H4
UFM1-H4
H4
UBA5
UFC1
UFL1
e
HA-WT H4
HA-K31R H4
HA
alpha tubulin
H3
f
Purified Nucleosome
WT
K31R
200 bp
100 bp
agarose gel
HA
g
WT
K31R
GFP Positive cells (%)
HR
h
GFP Positive cells (%)
NHEJ
i
Cell Cycle Distribution (%)
WT H4
K31R H4
G1
G2/M
S
j
Colony formation (%)
WT H4+Control siRNA
K31R H4+Control siRNA
WT H4+ATM siRNA
K31RH4+ATM siRNA
Gy
k
Phospho-H3 Positive cells (%)
H4 WT+Ctrl i
H4K31R+Ctrl i
H4WT+ATMi
H4K31R+ATMi
l
Control shRNA
Cul4A shRNA1
Cul4A shRNA2
IR
pS1981 ATM
ATM
pT68 Chk2
Chk2
Cul4A
Actin

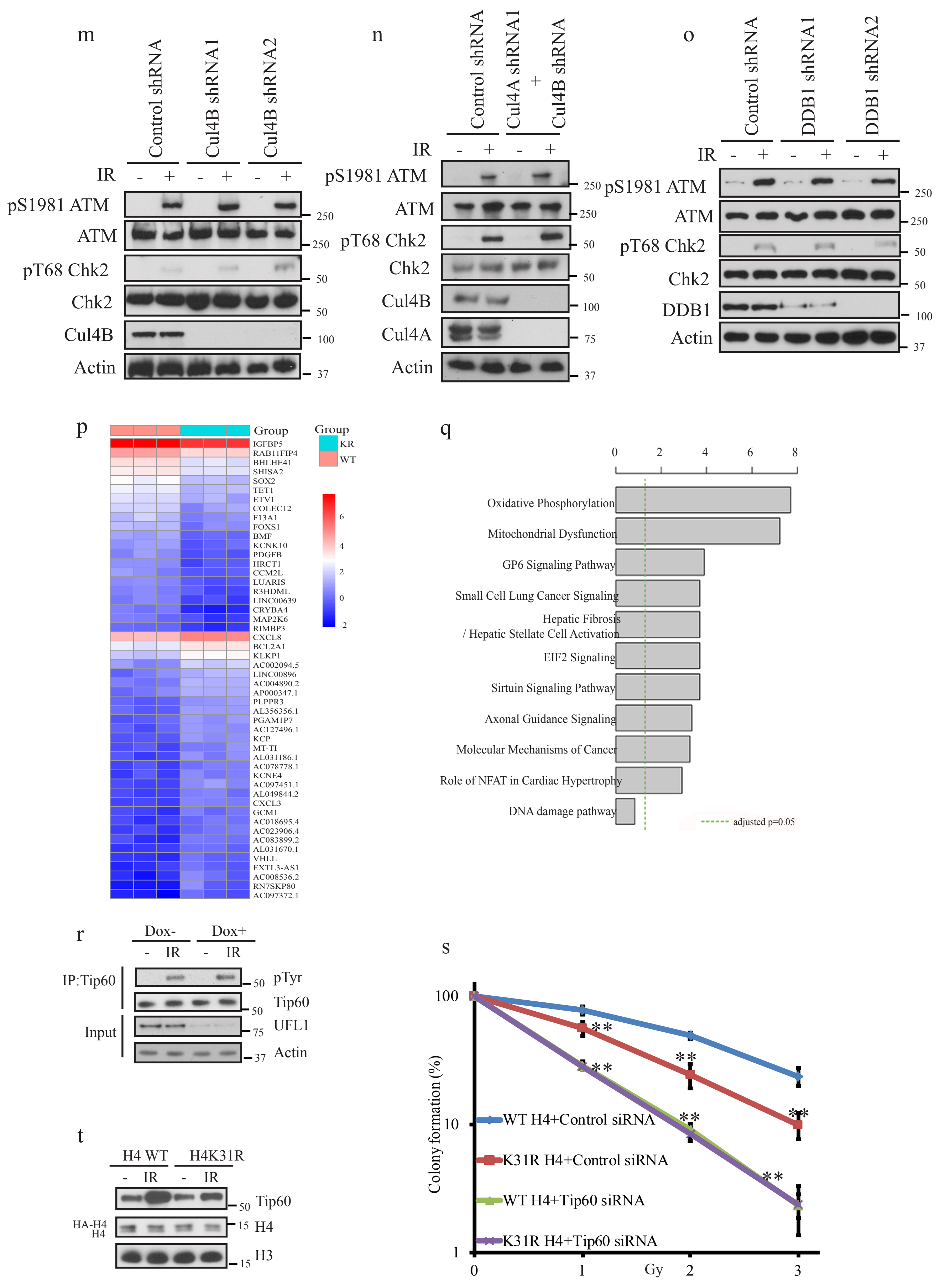
m
Control shRNA
Cul4B shRNA1
Cul4B shRNA2
IR - + - + - +
pS1981 ATM
ATM
pT68 Chk2
Chk2
Cul4B
Actin
250
250
50
50
100
37
n
Control shRNA
Cul4A shRNA1 + Cul4B shRNA
IR - + - +
pS1981 ATM
ATM
pT68 Chk2
Chk2
Cul4B
Cul4A
Actin
250
250
50
50
100
75
37
o
Control shRNA
DDB1 shRNA1
DDB1 shRNA2
IR - + - + - +
pS1981 ATM
ATM
pT68 Chk2
Chk2
DDB1
Actin
250
250
50
50
100
37
p
Group
Group
KR
WT
IGFBP5
RAB11FIP4
BHLHE41
SHISA2
SOX2
TET1
ETV1
COLEC12
F13A1
FOXS1
BMF
KCNK10
PDGFB
HRCT1
CCM2L
LUARIS
R3HDML
LINC00639
CRYBA4
MAP2K6
RIMBP3
CXCL8
BCL2A1
KLKP1
AC002094.5
LINC00896
AC004890.2
AP000347.1
PLPPR3
AL356356.1
PGAM1P7
AC127496.1
KCP
MT-TI
AL031186.1
AC078778.1
KCNE4
AC097451.1
AL049844.2
CXCL3
GCM1
AC018695.4
AC023906.4
AC083899.2
AL031670.1
VHLL
EXTL3-AS1
AC008536.2
RN7SKP80
AC097372.1
6
4
2
0
-2
q
0
2
4
6
8
Oxidative Phosphorylation
Mitochondrial Dysfunction
GP6 Signaling Pathway
Small Cell Lung Cancer Signaling
Hepatic Fibrosis / Hepatic Stellate Cell Activation
EIF2 Signaling
Sirtuin Signaling Pathway
Axonal Guidance Signaling
Molecular Mechanisms of Cancer
Role of NFAT in Cardiac Hypertrophy
DNA damage pathway
adjusted p=0.05
r
Dox-
Dox+
- IR - IR
IP:Tip60
Input
pTyr
Tip60
UFL1
Actin
50
50
75
37
s
Colony formation (%)
100
10
1
0
1
2
3
Gy
WT H4+Control siRNA
K31R H4+Control siRNA
WT H4+Tip60 siRNA
K31R H4+Tip60 siRNA
**
t
H4 WT
H4K31R
- IR - IR
Tip60
HA-H4
H4
H4
H3
50
15
15

Supplementary Fig.3 UFL1 monoufmylates histone H4 and promotes ATM activation.

(**a**) His-ufmylated proteins were purified from untreated or irradiated 293T cells and detected with indicated antibodies. (**b-d**) *In vitro* ufmylation assay. Purified UBA5, UFC1, UFL1, and UFM1 were incubated with candidate histone (H2A, H2B, H3 or H4) proteins (**b**), recombinant nucleosome (**c**), or recombinant WT and K31R H4 protein (**d**) in the presence of ATP and $MgCl_2$ at 30°C for 90 min. Ufmylation of histone was then examined by Western blot. (**e**) WT and K31R H4 plasmids were transfected into U2OS cells. 24 hours later, the cells were harvested and fractionated by chromatin fractionation buffer. The samples were blotted with indicated antibodies. (**f**) Purified nucleosome was analyzed by 1.5% agarose gel and western blot with indicated antibodies. **(g-h)** U2OS cells integrated with HR or NHEJ reporter and infected with UFL1 Tet-on shRNA1 and shRNA2 virus were transfected with wildtype (WT) and K31R H4 separately and subjected to the HR assay and NHEJ assay as described in the method. Data presented as mean ± SD of n=3 biological triplicates. **P<0.01. Statistical significance was calculated using 2-way student t-test (**i**) Cell cycle distribution of cells expressing WT histone H4 or the K31R mutant. Results (mean ± SD) are from three experiments. (**j**) Colony formation of U2OS cells expressing wildtype (WT) or K31R H4 and treated with or without ATM siRNA. The data presented are mean ± s.e.m. for n=3 independent experiments.** indicates p<0.01. Statistical significance was calculated using 2-way ANOVA test (**k**) Analysis of phospho-H3 positive U2OS cells expressing wildtype (WT) and K31R H4 and treated with or without ATM siRNA. The data presented are mean ± s.e.m. for n=3 independent experiments. ** indicates p<0.01. Statistical significance was calculated using 2-way ANOVA test (**1-o**) ATM signaling after ±IR in cells expressing Cul4A shRNA (**l**), Cul4B shRNA (**m**), Cul4A shRNA+ Cul4B shRNA (**n**), or DDB1 shRNA (**o**). (**p**) Differential gene expression in WT and K31R H4 expressing U2OS cells. (**q**) Pathway analysis of changed genes in WT and K31R H4 expressing U2OS cells. (**r**) U2OS cells stably expressing UFL1 Tet-on shRNA sh1 were treated with or without doxycycline. 30 minutes after 2 Gy IR, the cell lysates were incubated with Tip60 antibody and immunoprecipitates were blotted with indicated antibodies. (**s**) Colony formation of U2OS cells expressing wildtype (WT) and K31R H4 were treated with or without TIP60 siRNA. The data presented are mean ± s.e.m. for n=3 independent experiments. ** indicates p<0.01. Statistical significance was calculated using 2-way ANOVA. (**t**) U2OS cells expressing wildtype (WT) and K31R H4 were treated with 10 Gy IR and chromatin fraction was isolated and blotted with indicated antibodies.

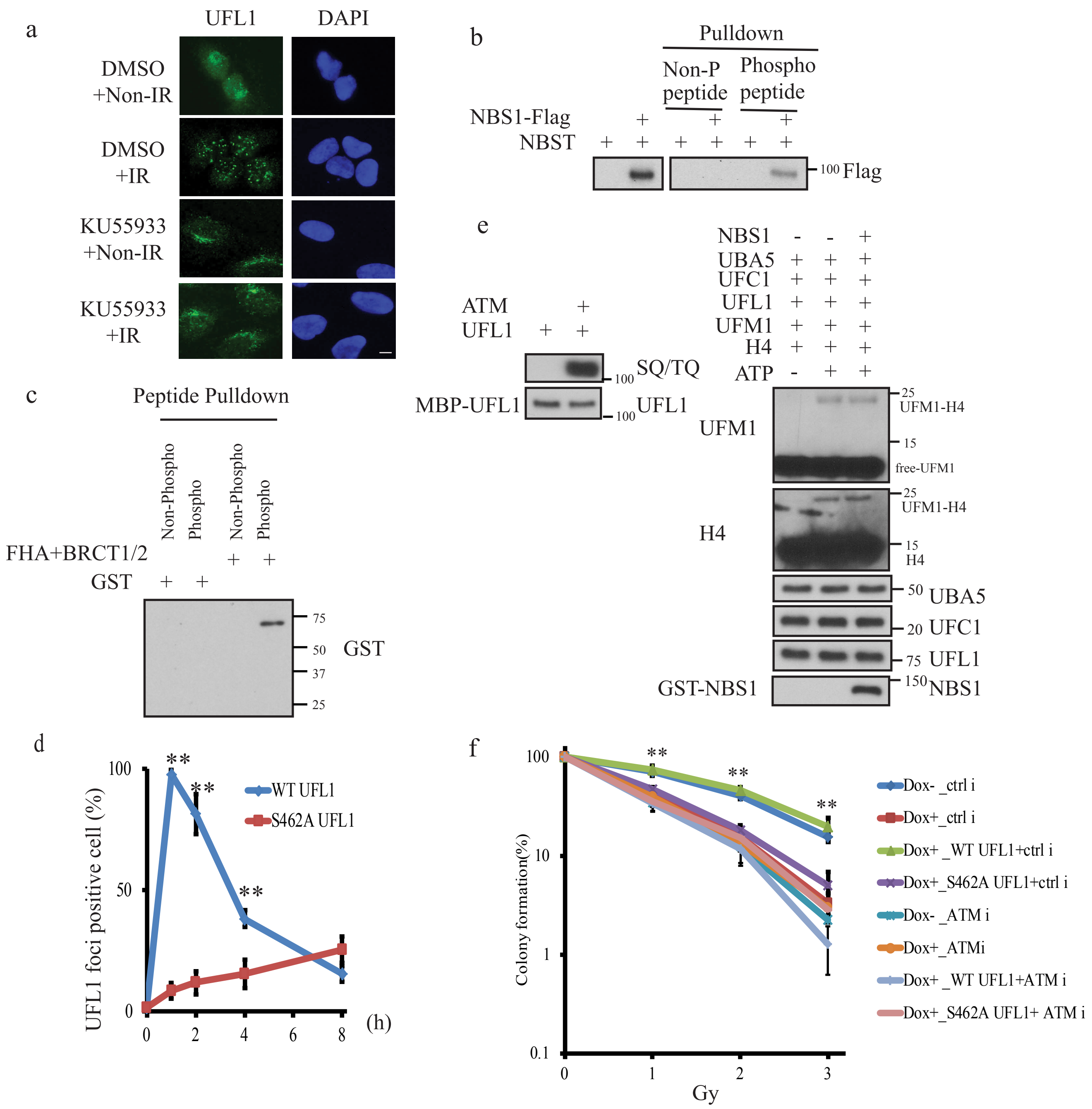
a
UFL1
DAPI
DMSO +Non-IR
DMSO +IR
KU55933 +Non-IR
KU55933 +IR
b
Pulldown
Non-P peptide
Phospho peptide
NBS1-Flag
NBST
100
Flag
c
Peptide Pulldown
Non-Phospho
Phospho
Non-Phospho
Phospho
FHA+BRCT1/2
GST
75
50
37
25
GST
e
ATM
UFL1
SQ/TQ
MBP-UFL1
UFL1
100
NBS1
UBA5
UFC1
UFL1
UFM1
H4
ATP
UFM1
25
UFM1-H4
15
free-UFM1
H4
25
UFM1-H4
15
H4
50
UBA5
20
UFC1
75
UFL1
GST-NBS1
150
NBS1
d
UFL1 foci positive cell (%)
100
50
0
0
2
4
6
8
(h)
**
WT UFL1
S462A UFL1
f
Colony formation(%)
100
10
1
0.1
0
1
2
3
Gy
**
Dox- _ctrl i
Dox+_ctrl i
Dox+ _WT UFL1+ctrl i
Dox+_S462A UFL1+ctrl i
Dox- _ATM i
Dox+_ATMi
Dox+ _WT UFL1+ATM i
Dox+_S462A UFL1+ ATM i

Supplementary Fig.4. ATM phosphorylates UFL1 at S462, and enhances its activity

(**a**) Immunostaining of UFL1 in U2OS cells treated with IR and or ATM inhibitor KU55933. (**b**) NBST cells were transfected with vector or Flag-tagged NBS1- full length plasmid. Cells were then lysed and incubated with biotinylated nonphosphorylated (non-P) or phosphorylated (phospho)-UFL1 peptide. The precipitates were analyzed with indicated antibody. Scale bars, 10 µm. (**c**) Purified GST, GST-FHA+BRCT1/2 NBS1 (was incubated with biotinylated nonphospho or phospho-UFL1 peptide separately. The precipitates were analyzed with indicated antibodies. (**d**) Quantification of WT and S462A UFL1 foci positive cells. Mean ± s.e.m. are from n=3 experiments. ** indicates $p<0.01$. Statistical significance was calculated using 2-way ANOVA test (**e**) Purified recombinant UFL1 protein was incubated with ATM protein, which was immunoprecipitated from U2OS cells, and phosphorylation of UFL1 protein was confirmed by phospho-SQ/TQ antibody(left panel). Purified UBA5, UFC1, and UFM1 and phosphorylated UFL1 protein were incubated with or without NBS1 purified proteins in the presence of ATP and $MgCl_2$ at 30°C for 90 min. Ufmylation of histone was then examined by Western blot. (Right panel) (**f**) Colony formation of control, UFL1 knockdown cells, or UFL1 knockdown cells reconstituted with WT or S462A mutant UFL1 and/or ATM siRNA. Mean ± s.e.m. are from n=3 experiments. ** indicates $p<0.01$. Dots depict individual data points. Statistical significance was calculated using 2-way ANOVA test.

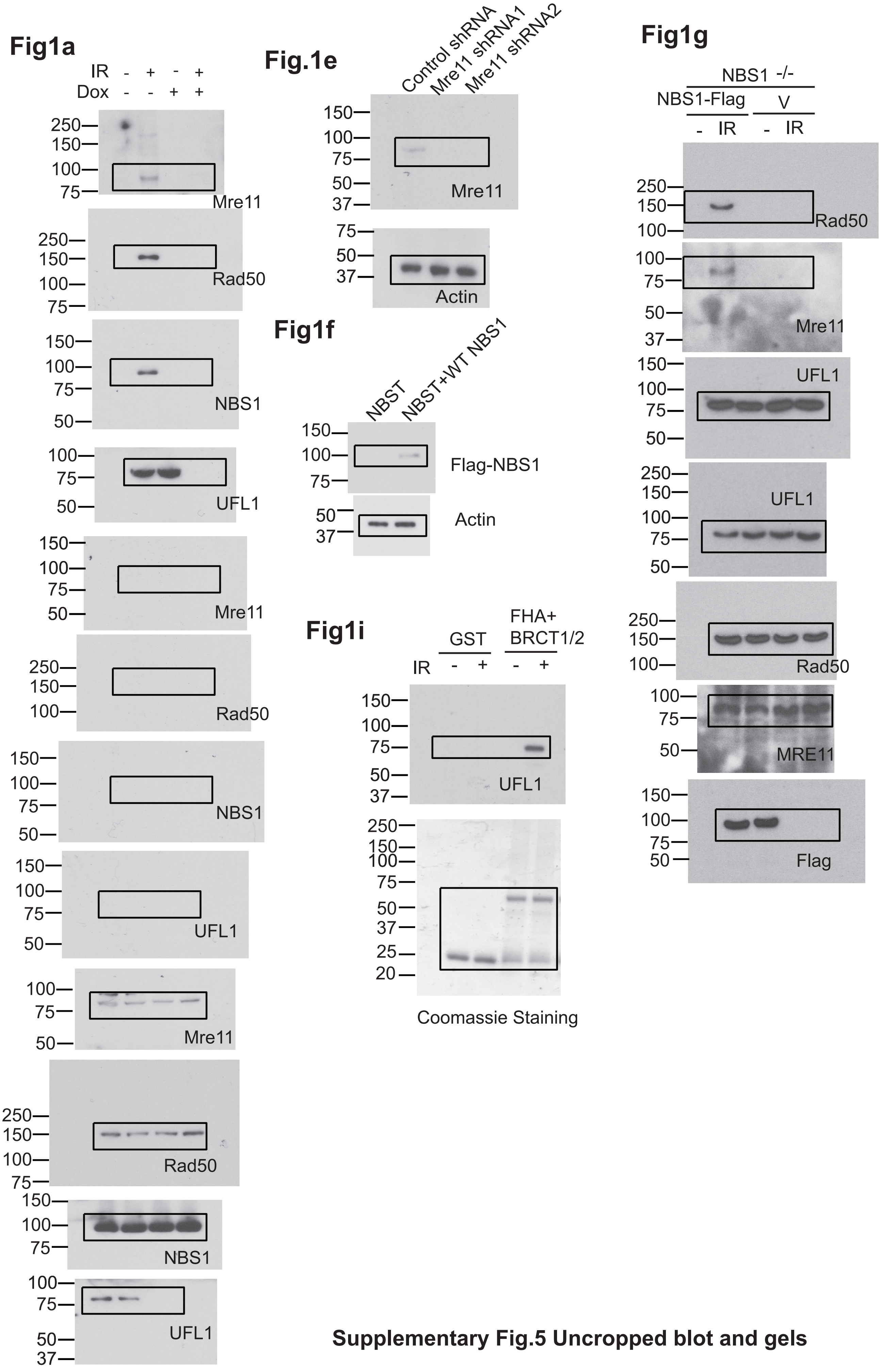

Supplementary Fig.5 Uncropped blot and gels

Fig.2a

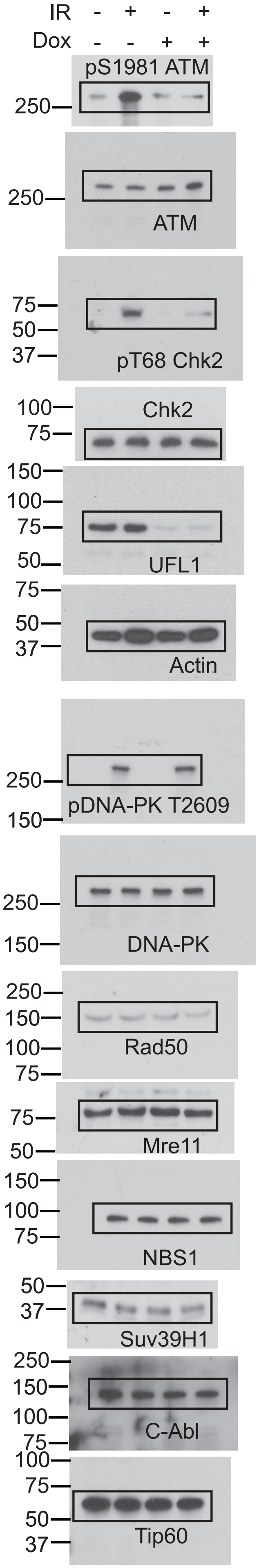

Fig.3g

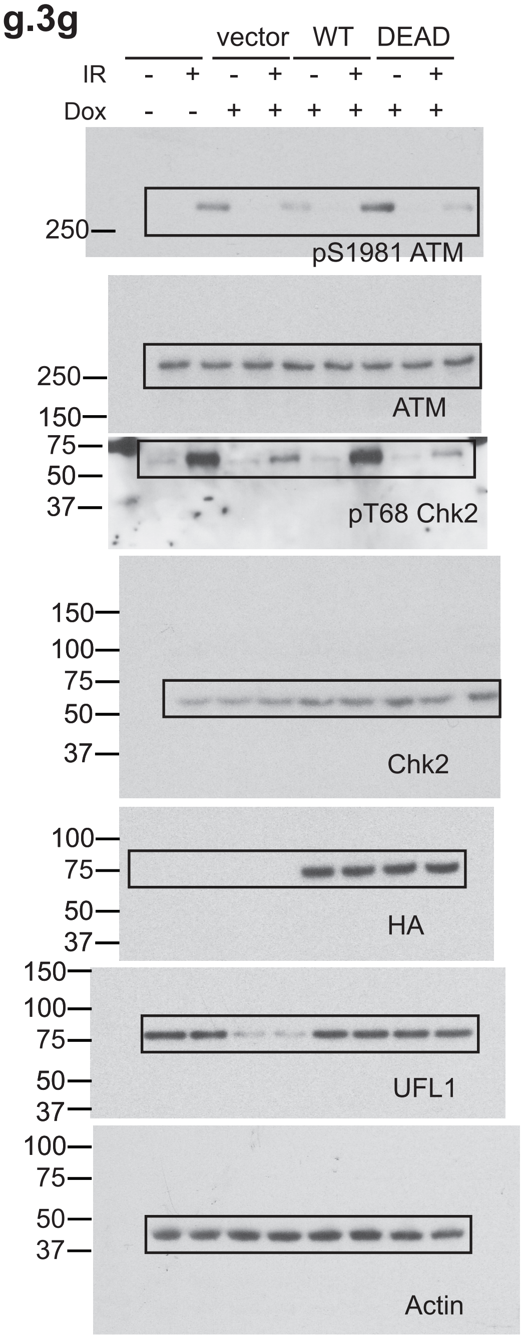

Fig.3h

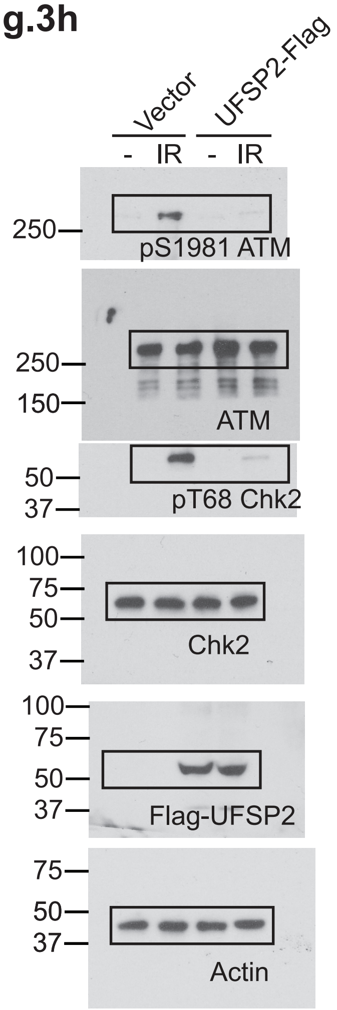

Supplementary Fig.5 Uncropped blot and gels

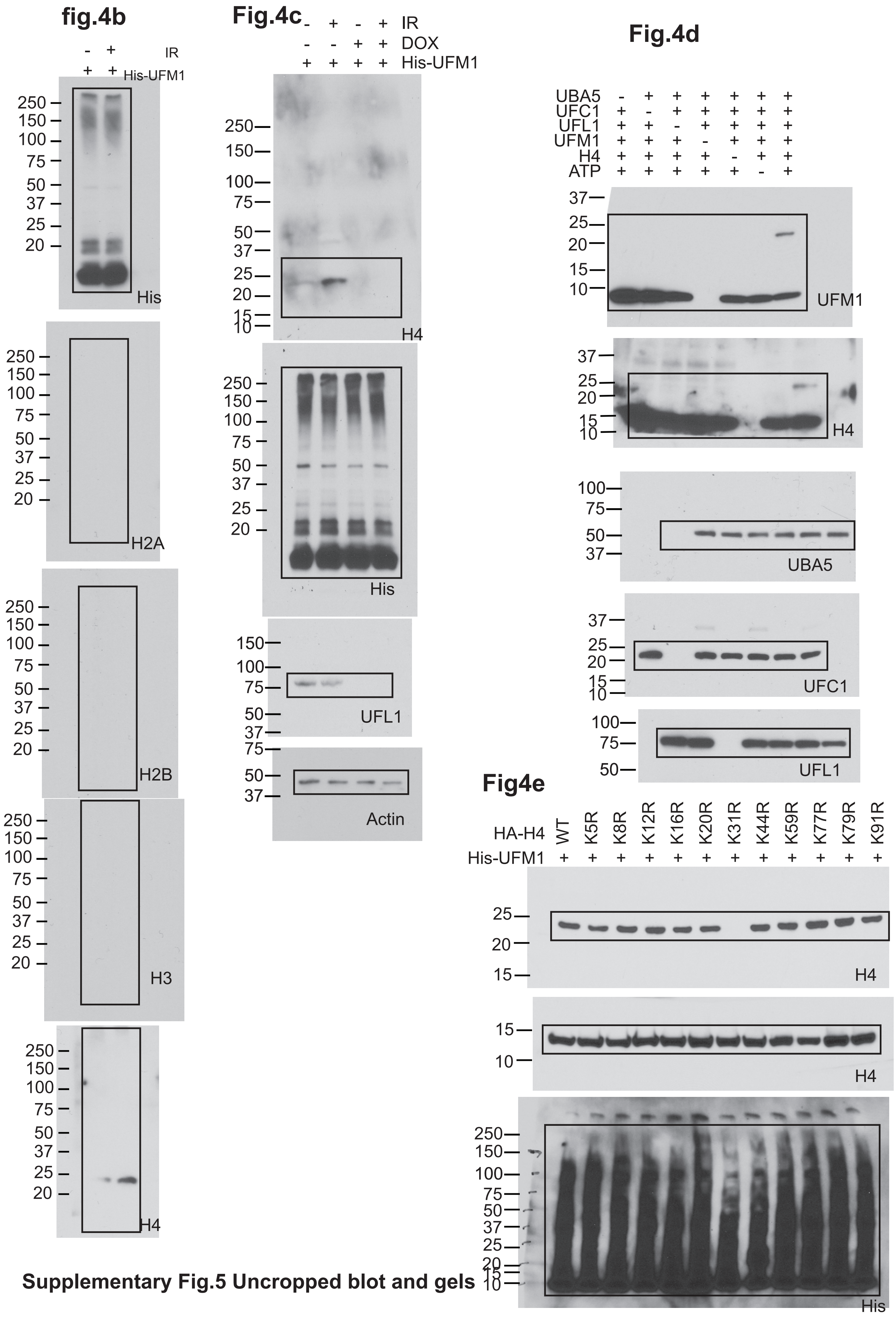

Supplementary Fig.5 Uncropped blot and gels

Fig.4f

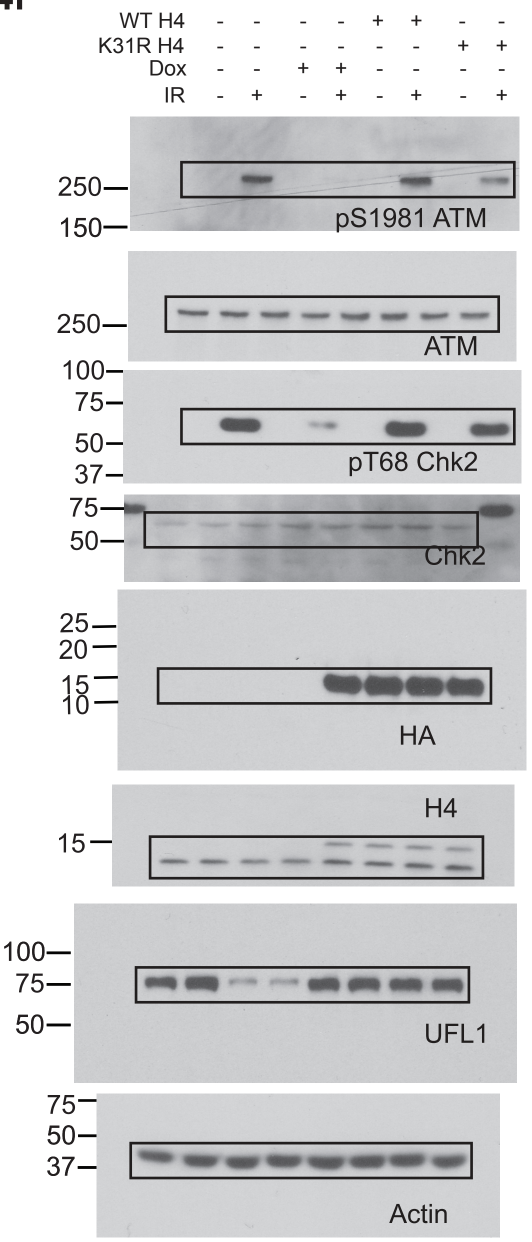

Fig4h

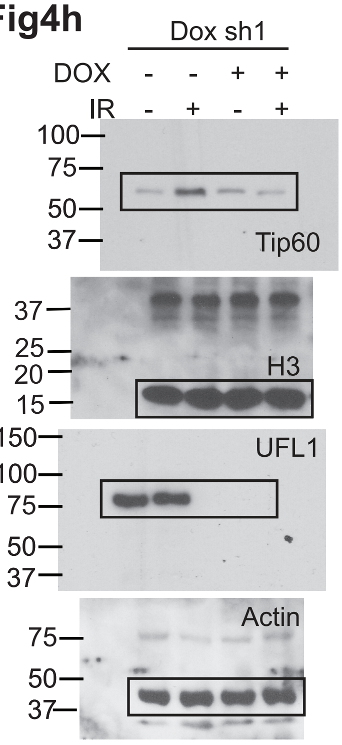

Fig6a

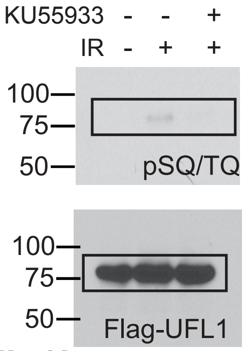

Fig6b

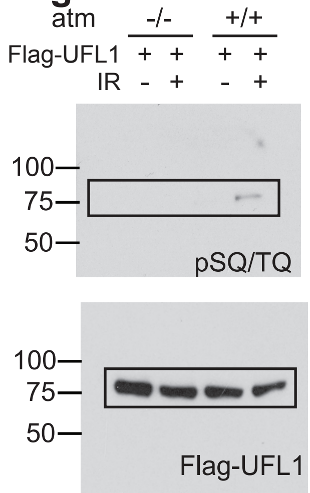

Fig6c

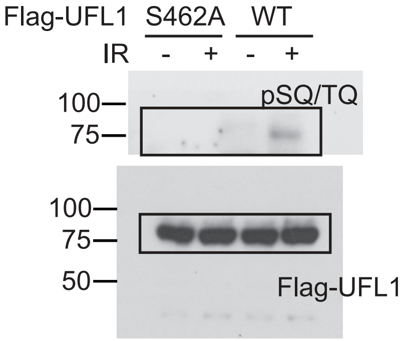

Supplementary Fig.5 Uncropped blot and gels

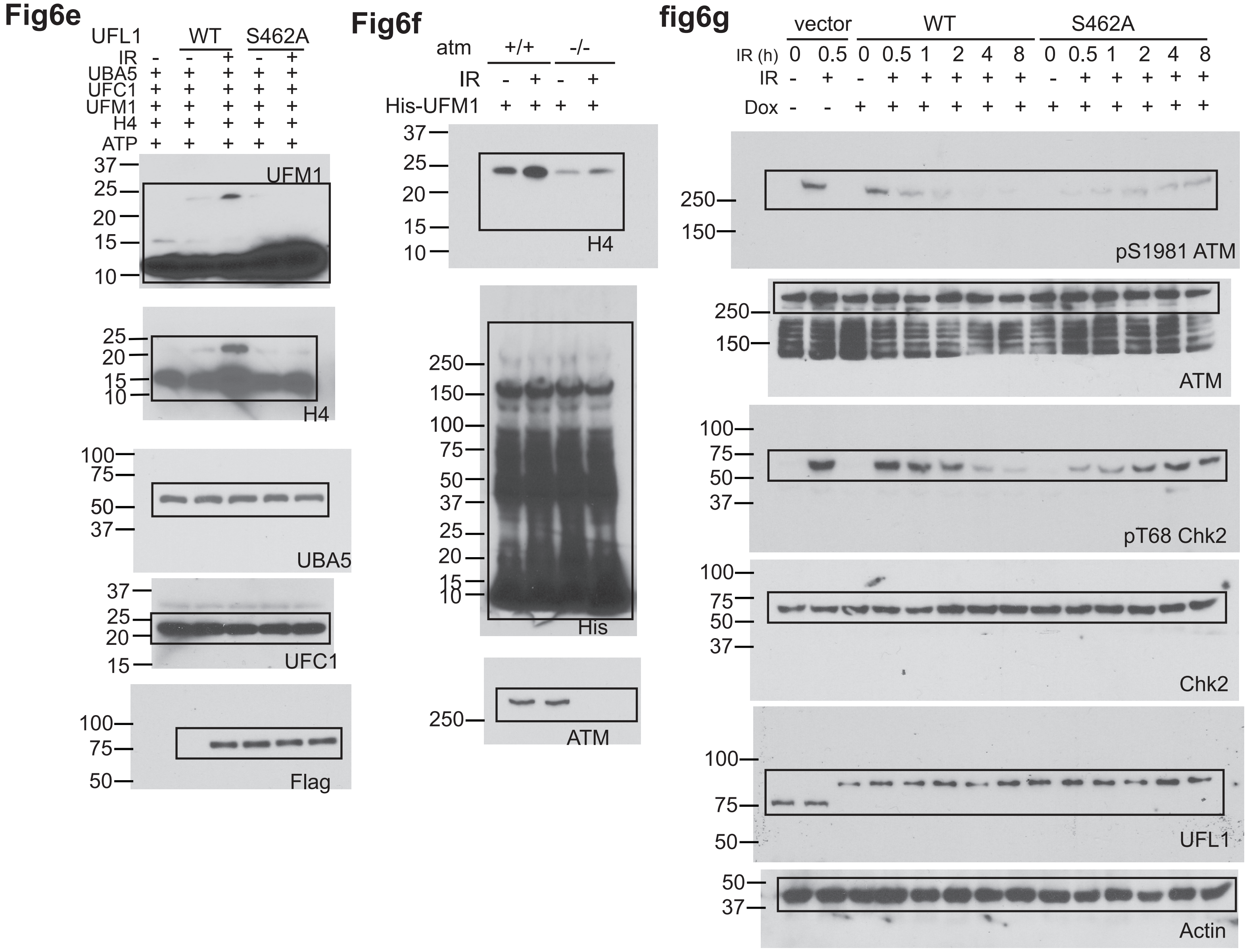

Supplementary Fig.5 Uncropped blot and gels

Supplementary fig1a

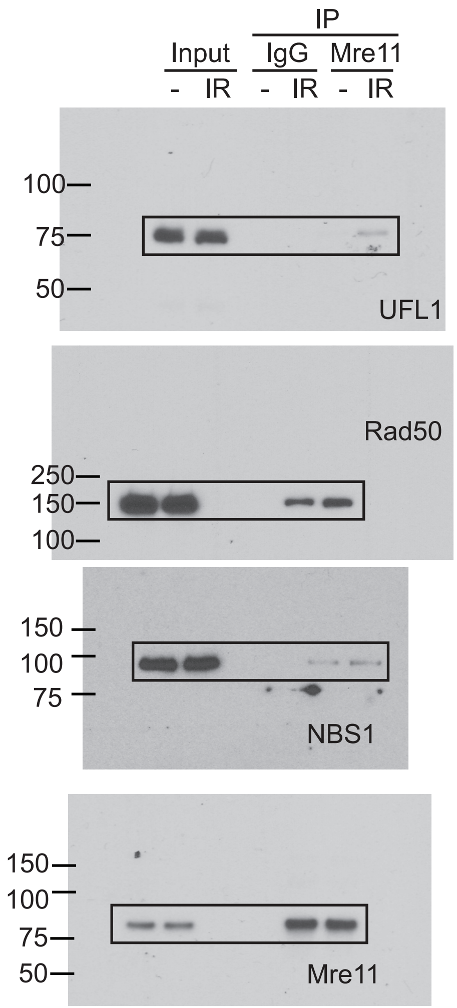

Supplementary fig1d

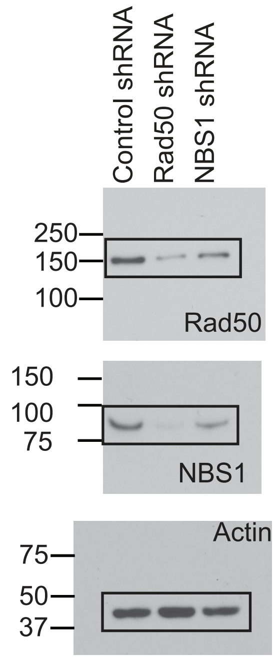

Supplementary fig1g

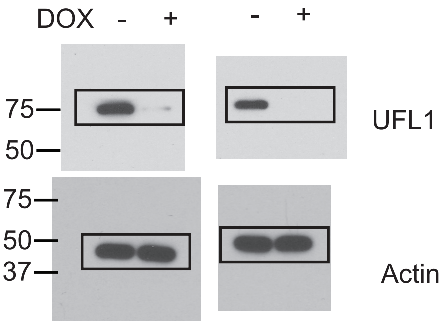

Supplementary fig2a

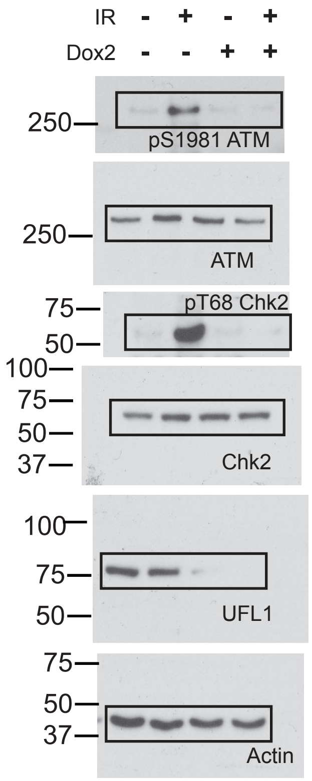

Supplementary Fig.5 Uncropped blot and gels

Supplementary fig2b

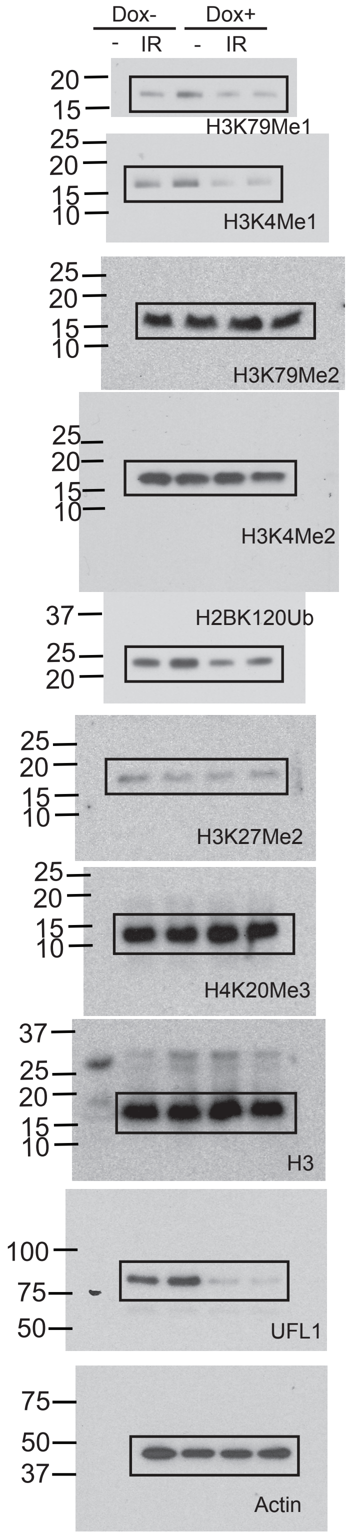

Supplementary Fig2l

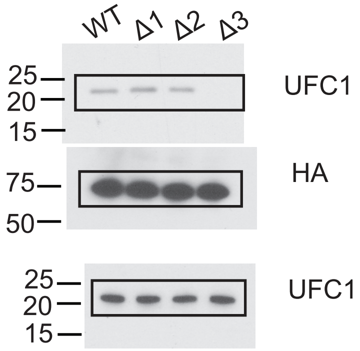

Supplementary Fig2n &o

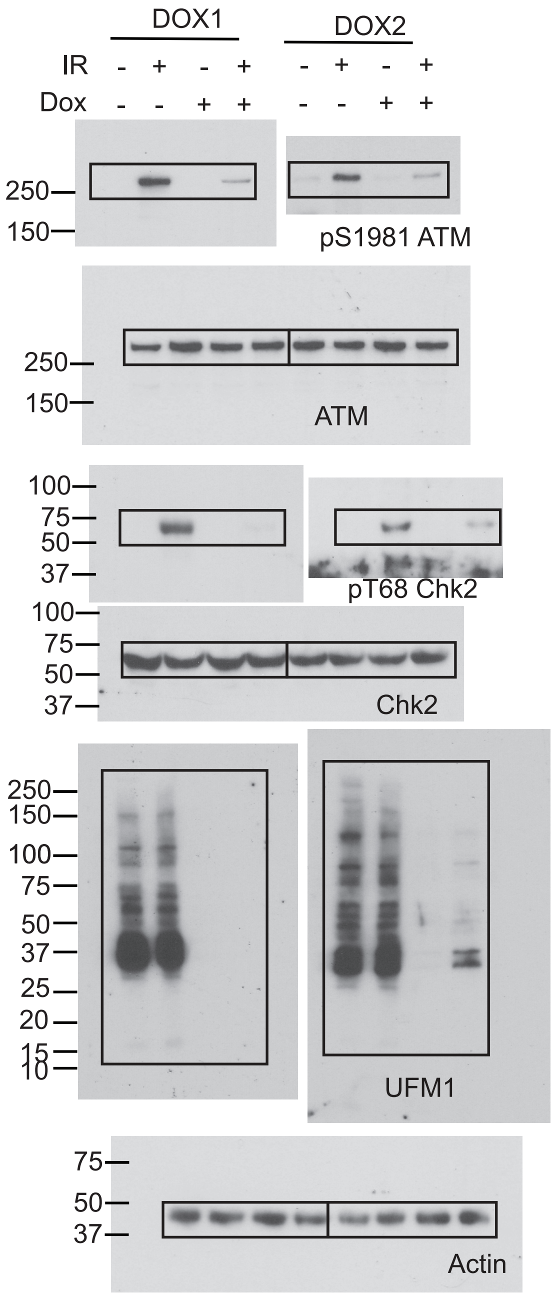

Supplementary Fig.5 Uncropped blot and gels

Supplementary fig3a

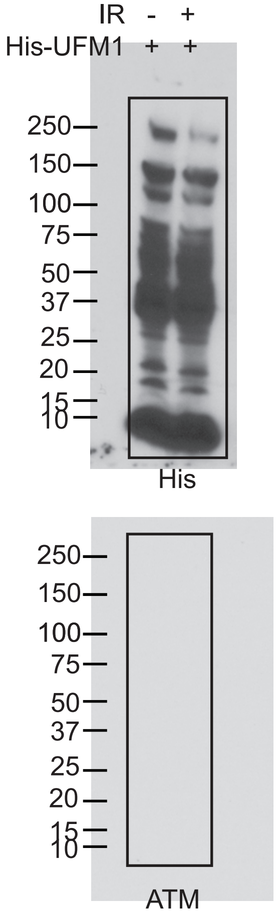

Supplementary fig.3b

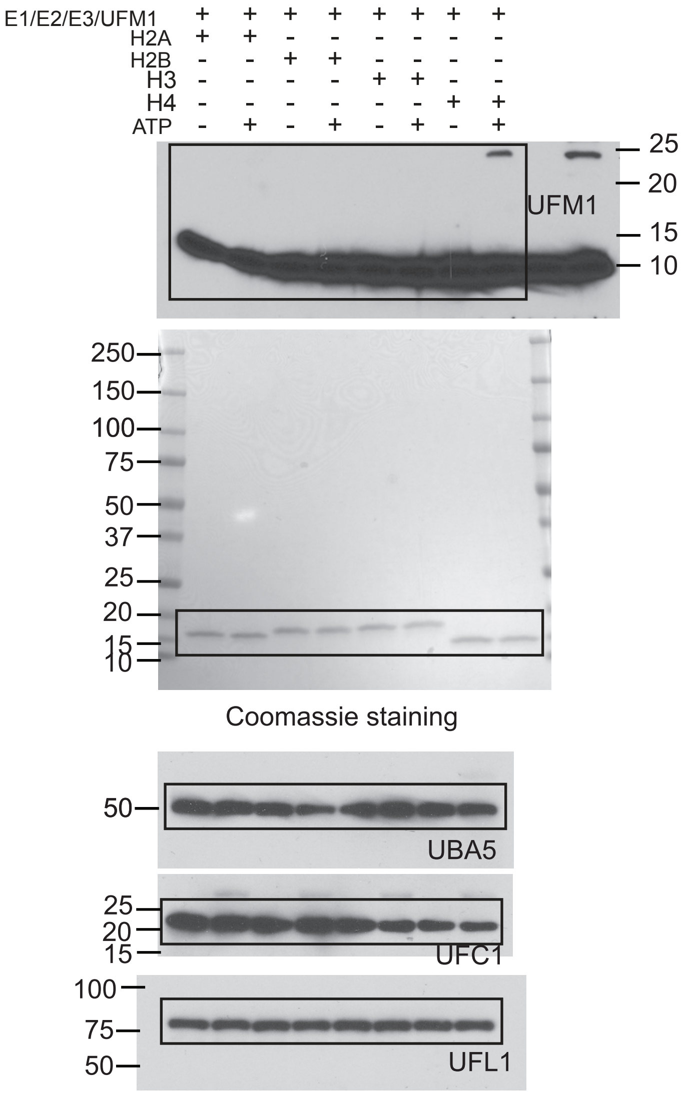

Supplementary Fig.5 Uncropped blot and gels

Supplementary Fig3c

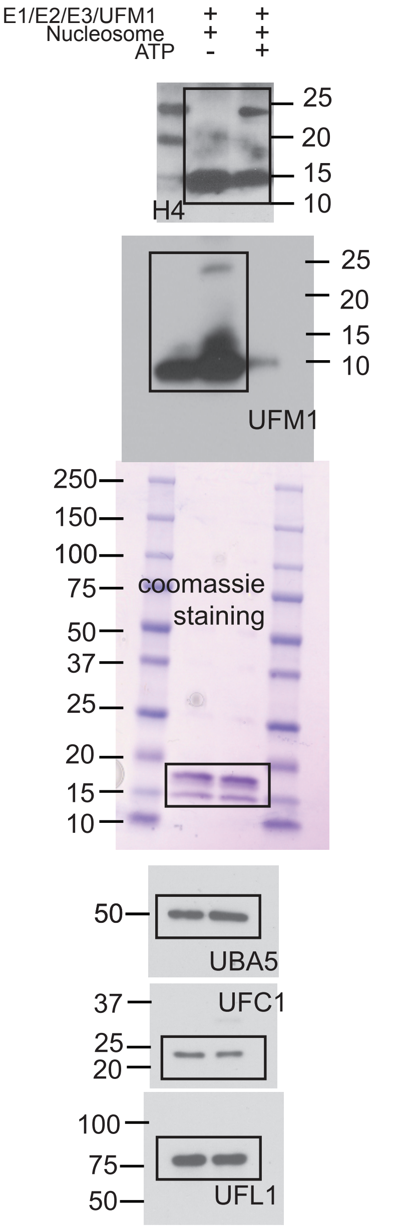

Supplementary Fig3d

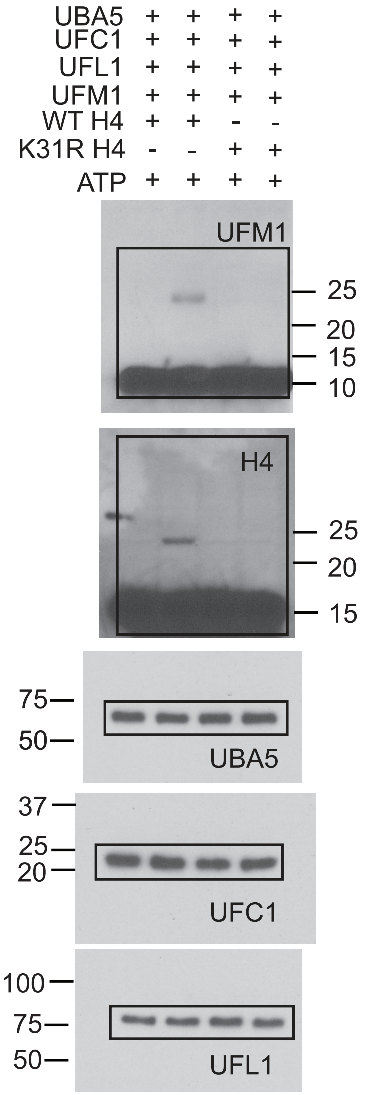

Supplementary Fig3e

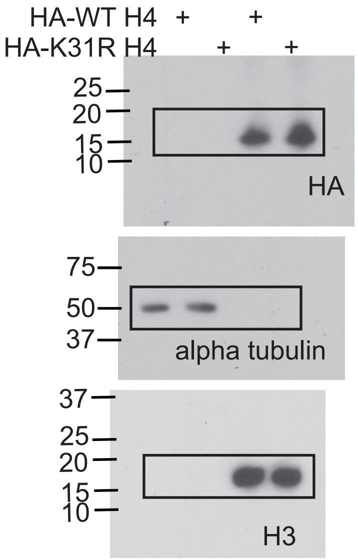

Supplementary Fig3f

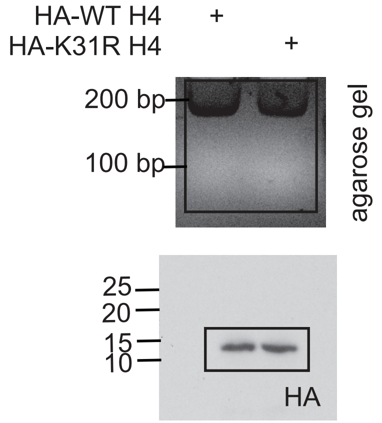

Supplementary Fig.5 Uncropped blot and gels

Supplementary fig3l

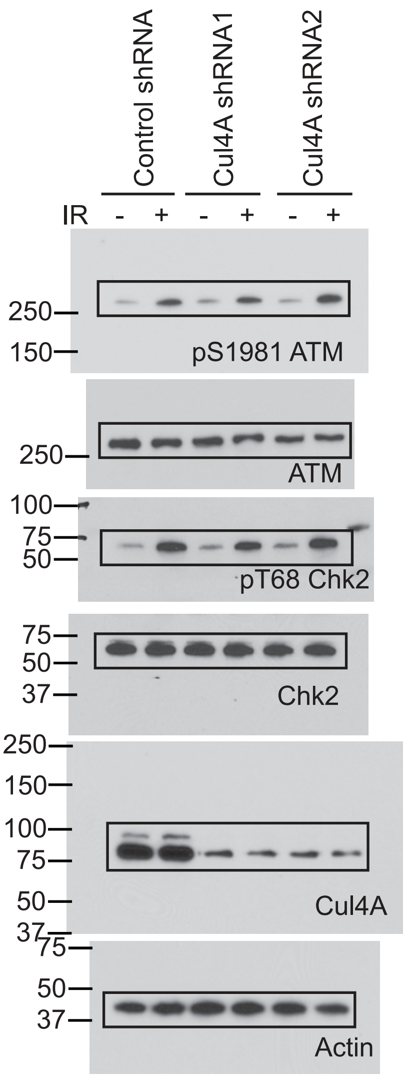

Supplementary fig3m

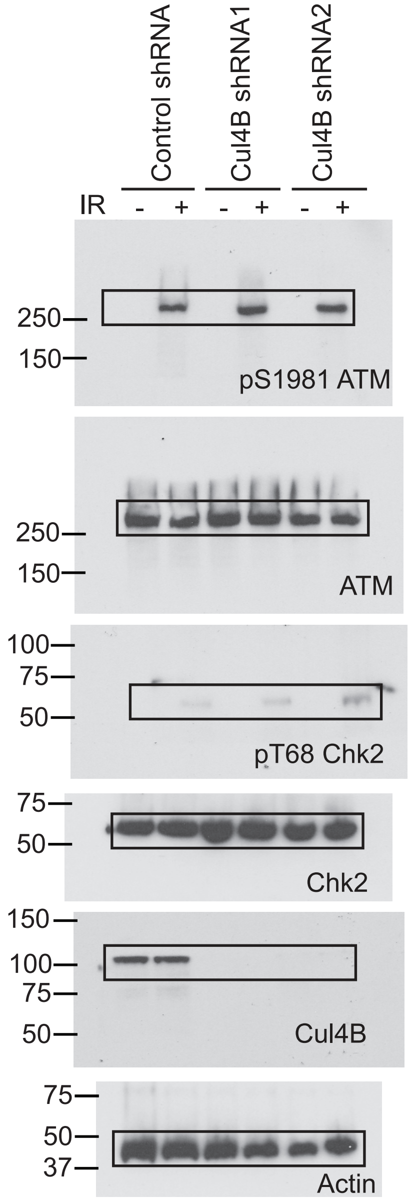

Supplementary fig3n

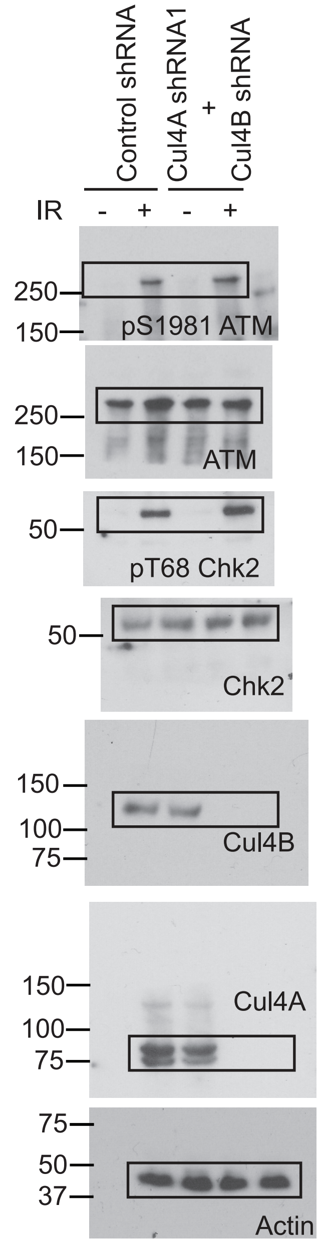

Supplementary fig3r

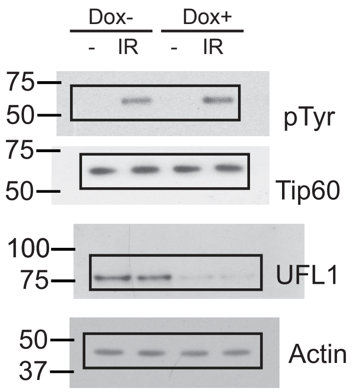

Supplementary fig3t

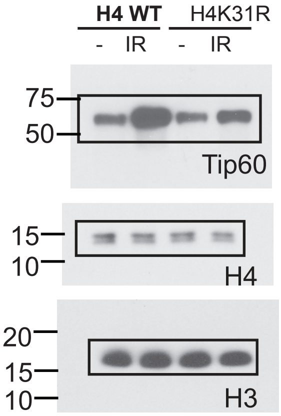

Supplementary Fig.5 Uncropped blot and gels

Supplementary Fig3o

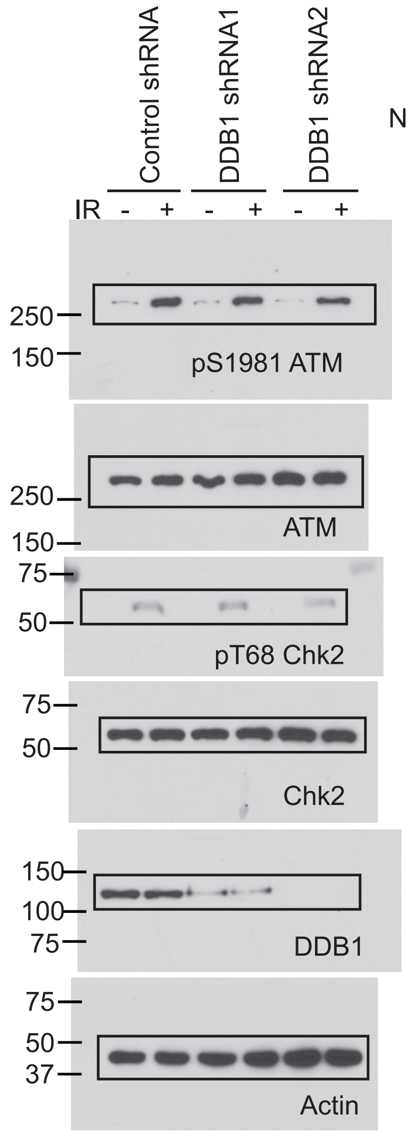

Supplementary fig.4b

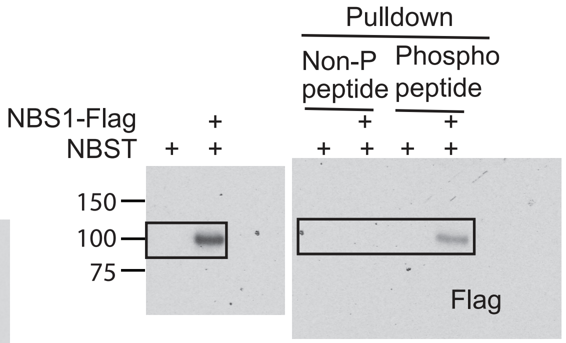

Supplementary fig.4c

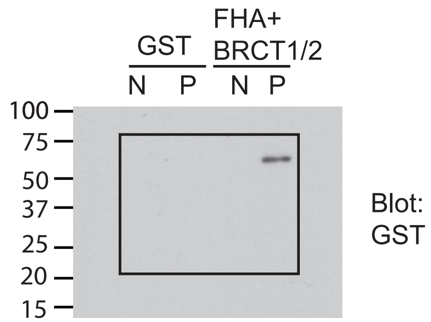

Supplementary fig.4e

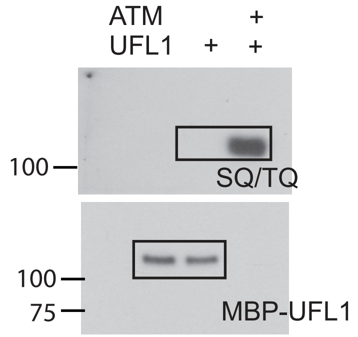

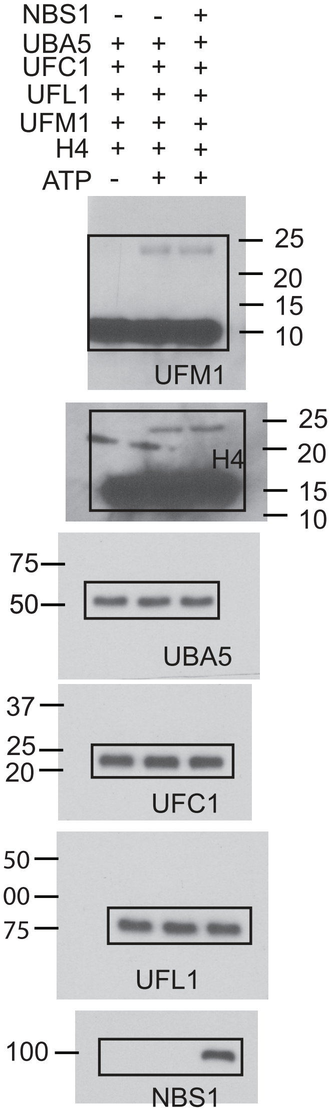

Supplementary Fig.5 Uncropped blot and gels
