# Supplementary material for: UFL1 promotes histone H4 ufmylation and ATM activation
Source: Nat Commun. 2019 Mar 18;10:1242. doi: 10.1038/s41467-019-09175-0 (PMC6423285; doi:10.1038/s41467-019-09175-0)
Supplement: Supplementary file 2 — Description of Additional Supplementary Files [file 41467_2019_9175_MOESM2_ESM.docx]

**Description of Supplementary Files**

**File Name:** Supplementary Data 1.

**Description:** Mass spectrometry analysis of UFM1 modified proteins. Flag-His vector or Flag-His-UFM1 transfected cells were purified with Nickel beads and Flag beads. The binding protein were eluted off with Flag peptide and analyzed by Mass spectrometry. The unique peptide and total peptide number for each protein, protein name, average signal intensity, gene symbol and molecular weight were listed in the table.
