# Supplementary material for: UFL1 promotes histone H4 ufmylation and ATM activation
Source: Nat Commun. 2019 Mar 18;10:1242. doi: 10.1038/s41467-019-09175-0 (PMC6423285; doi:10.1038/s41467-019-09175-0)
Supplement: Supplementary file 4 — Reporting Summary [file 41467_2019_9175_MOESM4_ESM.pdf]

nature research

Corresponding author(s): Zhenkun Lou

Last updated by author(s): Feb 12, 2019

Reporting Summary

Nature Research wishes to improve the reproducibility of the work that we publish. This form provides structure for consistency and transparency in reporting. For further information on Nature Research policies, see Authors & Referees and the Editorial Policy Checklist.

Statistics

For all statistical analyses, confirm that the following items are present in the figure legend, table legend, main text, or Methods section.

n/a	Confirmed	
☐	☒	The exact sample size (*n*) for each experimental group/condition, given as a discrete number and unit of measurement
☐	☒	A statement on whether measurements were taken from distinct samples or whether the same sample was measured repeatedly
☐	☒	The statistical test(s) used AND whether they are one- or two-sided<br>*Only common tests should be described solely by name; describe more complex techniques in the Methods section.*
☒	☐	A description of all covariates tested
☐	☒	A description of any assumptions or corrections, such as tests of normality and adjustment for multiple comparisons
☐	☒	A full description of the statistical parameters including central tendency (e.g. means) or other basic estimates (e.g. regression coefficient) AND variation (e.g. standard deviation) or associated estimates of uncertainty (e.g. confidence intervals)
☐	☒	For null hypothesis testing, the test statistic (e.g. *F*, *t*, *r*) with confidence intervals, effect sizes, degrees of freedom and *P* value noted<br>*Give P values as exact values whenever suitable.*
☒	☐	For Bayesian analysis, information on the choice of priors and Markov chain Monte Carlo settings
☒	☐	For hierarchical and complex designs, identification of the appropriate level for tests and full reporting of outcomes
☒	☐	Estimates of effect sizes (e.g. Cohen's *d*, Pearson's *r*), indicating how they were calculated

Our web collection on statistics for biologists contains articles on many of the points above.

Software and code

Policy information about availability of computer code

Data collection	Cell Quest was used to collect flow cytometry data.
Data analysis	GraphPad Prism (version 7) and Flow Jo (version 10) were used to analyze the data.<br>Please refer to methods section for details. STAR aligner (v2.3.0e) is used for RNA seq alignment. limma for estimating the mean and variance relationship. Canonical functional pathways enriched in the differentially expressed genes (DEGs) were identified through the use of Ingenuity Pathway Analysis (IPA) (QIAGEN Inc.)

For manuscripts utilizing custom algorithms or software that are central to the research but not yet described in published literature, software must be made available to editors/reviewers. We strongly encourage code deposition in a community repository (e.g. GitHub). See the Nature Research guidelines for submitting code & software for further information.

Data

Policy information about availability of data

All manuscripts must include a data availability statement. This statement should provide the following information, where applicable:

- Accession codes, unique identifiers, or web links for publicly available datasets
- A list of figures that have associated raw data
- A description of any restrictions on data availability

All data are available from the corresponding author upon reasonable request. There are no restrictions on data availability. Raw data for each figure has been provided as Supplemental figures or tables. RNA-seq data used in Supplementary Fig.3p-q are deposited in a GEO database. The accession number is GSE126451.

Field-specific reporting

Please select the one below that is the best fit for your research. If you are not sure, read the appropriate sections before making your selection.

☒ Life sciences ☐ Behavioural & social sciences ☐ Ecological, evolutionary & environmental sciences

For a reference copy of the document with all sections, see nature.com/documents/nr-reporting-summary-flat.pdf

Life sciences study design

All studies must disclose on these points even when the disclosure is negative.

Sample size	No statistical method was used to predetermine sample size.
Data exclusions	No data were excluded from the analyses.
Replication	At least 3 independent experiments were performed for all . All findings were reliably reproduced.
Randomization	Samples were randomly allocated into experimental groups prior to treatment. For example, since most of our experiments involved cells, plates were randomly allocated to groups.
Blinding	One individual performed the experiment while another individual (blinded to the group allocation) performed the analysis.

Reporting for specific materials, systems and methods

We require information from authors about some types of materials, experimental systems and methods used in many studies. Here, indicate whether each material, system or method listed is relevant to your study. If you are not sure if a list item applies to your research, read the appropriate section before selecting a response.

Materials & experimental systems

n/a	Involved in the study	
☐	☒	Antibodies
☐	☒	Eukaryotic cell lines
☒	☐	Palaeontology
☒	☐	Animals and other organisms
☒	☐	Human research participants
☒	☐	Clinical data

Methods

n/a	Involved in the study	
☒	☐	ChIP-seq
☐	☒	Flow cytometry
☒	☐	MRI-based neuroimaging

Antibodies

Antibodies used	Anti-UFL1 antibody (A303-456A) for western blot, anti-NBS1 antibody (A301-284A for western) anti-DDB1 (A300-462A for western) and anti-Cul4B (A303-864A for western) were purchased from Bethyl. Anti-actin (A5316), anti-UFL1 (HPA030558) for detecting foci, anti-α tubulin (DM1A for western) and anti-STK38 (SAB1408832) antibodies for detecting foci were purchase from Sigma. Anti-ATM (2873 for western), anti-pSer1981 ATM (13050 for western), anti-Mre11 (4847 for western), anti-Rad50 (3427 for western), anti-Suv39h1 (8729), anti-Cul4A (2669 for western), anti-SQ/TQ motif (9607 for western), anti-Chk2 (2662 for western), anti-phosphotyrosine (9411 for western), anti-DNAPK (4602 for western), anti-H3K79me1 (12522 for western), anti-H3K4me1 (4326 for western), anti-H3K4me2 (9325 for western), anti-H2BK120ub (5546 for western), anti-H3K27me2 (9728 for western), anti-H4K20me3 (5737 for western) and anti-phosphoChk2 (2197 for western) antibodies were purchased from Cell Signaling. CTIP antibody (61141 for IF) was purchased from Active Motif. Anti-Tip60 (07-038 for western), anti-53BP1 (mab3802 for western and IF), anti-MDC1 (05-172), anti-Suv39h1 (MABE552 for western) and anti-γH2AX (05-636 for IF) antibodies were purchased from Millipore. Anti-UBA5 (ab177478 for western), anti-UFC1 antibodies (ab189252 for western), anti-H2A (ab18255 for western), anti-H2B (ab1790 for western), anti-H3 (ab1791 for Western and CHIP), anti-H4 (ab10158 for western and CHIP), anti-H4K16ac (ab109463 for CHIP), anti-H3K79me2 (ab3594, for western) and anti-H3K9me3 (ab8898) antibodies were purchased from Abcam. Anti-UFM1 (SC-84652 for IF and Western), anti-UFSP2 (sc-292068 for IF and Western), anti-RPA (sc-56770 for IF), anti-Rad51 (sc-8349 for IF) and anti-BRCA1 (sc-6954 for IF) antibodies were purchased from Santa Cruz. Rabbit 53BP1 antibody (NB100-304) and NBS1 antibody (NB100-143) for foci were purchased from Novus Biologicals.
Validation	Commercially available antibodies were validated by the supplier and by us using appropriate controls where needed. Please refer to the manufacturers' websites for details.

Eukaryotic cell lines

Policy information about cell lines

Cell line source(s)	U2OS, HEK293T, ROS8 MDA-MB-231
Authentication	The identities of all cell lines were confirmed by the Medical Genome facility at Mayo Clinic Center (Rochester, MN) using short tandem repeat profiling upon receipt.
Mycoplasma contamination	All cell lines were periodically tested for mycoplasma using MycoAlert™ Mycoplasma Detection Kit (Lonza, LT-07). None of the cell lines were contaminated.
Commonly misidentified lines (See ICLAC register)	No commonly misidentified cell lines were used.

Flow Cytometry

Plots

Confirm that:

☐ The axis labels state the marker and fluorochrome used (e.g. CD4-FITC).

☐ The axis scales are clearly visible. Include numbers along axes only for bottom left plot of group (a 'group' is an analysis of identical markers).

☐ All plots are contour plots with outliers or pseudocolor plots.

☐ A numerical value for number of cells or percentage (with statistics) is provided.

Methodology

Sample preparation	Cells were collected and spun down at 1000g. The samples were then fixed with formaldehyde, permeabilized as needed, and stained again (intracellular protein).
Instrument	Calibur (BD)
Software	Cell Quest and FlowJo
Cell population abundance	No flow based sorting was performed.
Gating strategy	Appropriate control samples were used to gate the samples. For cell cycle analysis, unstained cells were used as control. For phospho-H3 positive cells, the controls were unstained cell, PI only, phospho-H3 staining only and control IgG only stained cells. For HR or NHEJ reporter assay, the controls were unstained cells, GFP only cells, and mCherry only cells. After cells were selected in the FSC/SSC dot plot to remove debris, they were gated to exclude cellular aggregates in the FSC/FSC dot plot. Gates of positive cells were set and compared with a control sample (unstained cells) with no detectable fluorochrome expression.

☐ Tick this box to confirm that a figure exemplifying the gating strategy is provided in the Supplementary Information.
